# Time-domain observation of ballistic orbital-angular-momentum currents with giant relaxation length in tungsten

**DOI:** 10.1038/s41565-023-01470-8

**Published:** 2023-08-07

**Authors:** Tom S. Seifert, Dongwook Go, Hiroki Hayashi, Reza Rouzegar, Frank Freimuth, Kazuya Ando, Yuriy Mokrousov, Tobias Kampfrath

**Affiliations:** 1 Department of Physics, Freie Universität Berlin, Berlin, Germany; 2 Department of Physical Chemistry, Fritz-Haber-Institut der Max-Planck-Gesellschaft, Berlin, Germany; 3 Peter-Grünberg-Institut, Forschungszentrum Jülich, Jülich, Germany; 4 Institute of Physics, Johannes-Gutenberg-Universität Mainz, Mainz, Germany; 5 Department of Applied Physics and Physico-Informatics, Keio University, Yokohama, Japan; 6 Keio Institute of Pure and Applied Sciences, Keio University, Yokohama, Japan; 7 Center for Spintronics Research Network, Keio University, Yokohama, Japan

**Keywords:** Magnetic properties and materials, Spintronics, Terahertz optics

## Abstract

The emerging field of orbitronics exploits the electron orbital momentum *L*. Compared to spin-polarized electrons, *L* may allow the transfer of magnetic information with considerably higher density over longer distances in more materials. However, direct experimental observation of *L* currents, their extended propagation lengths and their conversion into charge currents has remained challenging. Here, we optically trigger ultrafast angular-momentum transport in Ni|W|SiO_2_ thin-film stacks. The resulting terahertz charge-current bursts exhibit a marked delay and width that grow linearly with the W thickness. We consistently ascribe these observations to a ballistic *L* current from Ni through W with a giant decay length (~80 nm) and low velocity (~0.1 nm fs^−1^). At the W/SiO_2_ interface, the *L* flow is efficiently converted into a charge current by the inverse orbital Rashba–Edelstein effect, consistent with ab initio calculations. Our findings establish orbitronic materials with long-distance ballistic *L* transport as possible candidates for future ultrafast devices and an approach to discriminate Hall-like and Rashba–Edelstein-like conversion processes.

## Abstract (web summary)

The observation of orbital currents with extended propagation lengths remains challenging. Here, Seifert et al. optically trigger ultrafast orbital currents in Ni|W|SiO_2_ stacks that seemingly propagate ballistically with a giant decay length and low velocity.

## Main

Electrons carry two distinct angular momenta: the spin angular momentum *S* and orbital angular momentum *L*. While *S* is successfully exploited in spintronics^1^ to transport information by *S* currents and to convert them into detectable charge (*C*) currents by spin-to-charge-current conversion (*SC*C)^2^, *L* has only recently gained attention in orbitronics research. To make this fascinating concept compatible and competitive with conventional electronics^3,4^, the speed of orbitronic functionalities needs to reach terahertz (THz) rates^5^.

A first key advantage of *L* over *S* is that *L* can assume arbitrarily high values per electron and, thus, enable efficient magnetic-order manipulation^1,6,7^ by *L*-induced torques^8–12^. Second, *L*-to-charge-current conversion (*LC*C) works in materials without spin–orbit interaction, including abundant light metals^13^. Finally, *L* currents are predicted to propagate over increased lengths of nearly 100 nm (ref. ^14^).

Recent studies provided strong indications of charge-to-*L* current conversion by the orbital Hall effect (OHE) in a thin layer of a paramagnetic material (PM). The resulting *L* current was typically interrogated through the torque it exerted on the magnetization of an adjacent thin-film ferromagnetic material (FM)^1,8–12,14–24^. The FM was chosen to be susceptible to either an *S* accumulation, for example, permalloy Ni_81_Fe_19_ (Py), or an *L* accumulation, for example, Ni.

Unfortunately, it remains experimentally challenging to measure *L* currents by *LC*C. First, it is difficult to distinguish *LC*C by the inverse OHE (IOHE) from *LC*C by an inverse orbital Rashba–Edelstein effect (IOREE) because both phenomena obey identical macroscopic symmetries. Second and for the same reason, IOHE and IOREE are difficult to separate from their *S* counterparts, that is, the inverse spin Hall effect (ISHE) and inverse spin Rashba–Edelstein effect (ISREE)^25^. Previous work, however, indicates different spatial propagation and relaxation dynamics of *S* and *L* currents^8,9,14^. Therefore, an experimental approach such as terahertz emission spectroscopy^26,27^, which monitors currents with femtosecond resolution, is perfectly suited to access the possibly different ultrafast *L*-versus-*S* propagation and conversion dynamics.

**Fig. 1:**
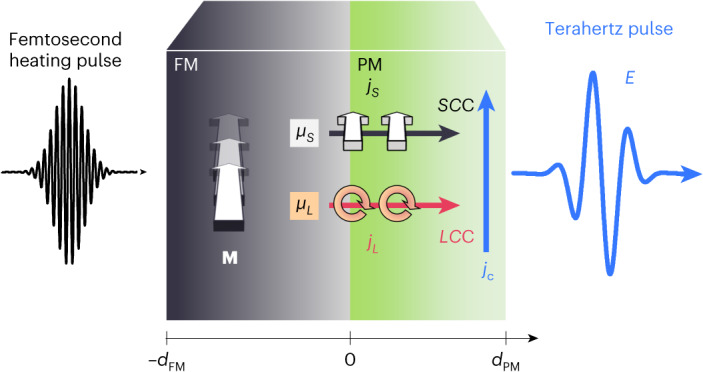
Launching and detecting terahertz *S* and *L* currents. Upon ultrafast laser excitation of the FM, an excess of FM magnetization **M** arises, leading to *S* accumulation *μ*_*S*_, *L* accumulation *μ*_*L*_ and the injection of spin and orbital currents *j*_*S*_ and *j*_*L*_, respectively, into the PM. Various bulk and interfacial *LC*C and *SC*C processes generate an ultrafast in-plane charge current *j*_*C*_ that emits a terahertz electromagnetic pulse with electric-field amplitude *E*(*t*) versus time *t* directly behind the sample.

Here, we study ultrafast signatures of *S* and *L* transport from an FM into a PM that is launched by exciting FM|PM stacks with a femtosecond laser pulse (Fig. 1). The *LC*C and *SC*C in the PM are measured by monitoring the emitted terahertz pulse. Upon changing the FM from Ni to Py and interfacing it with the PMs Pt, Ti and W, we find the same characteristic sign changes in the emitted terahertz field as in previous magneto-transport studies^9^. Consequently, we interpret our observations as hallmarks of ultrafast *LC*C and *SC*C.

Remarkably, the terahertz field from Ni|W is temporally strongly delayed and broadened relative to that from Ni|Pt. The bandwidth and amplitude of the underlying burst of charge current decreases with W thickness, whereas its delay and width increase linearly. We assign this observation to long-distance ballistic *L* transport in W, which has a relaxation length that is more than one order of magnitude larger than that of *S* transport. Specifically, our data and calculations suggest a dominant contribution to *LC*C through the IOREE at the W/SiO_2_ interface. This effect is absent in Ni|Ti and attributed to a dominant bulk *LC*C by the IOHE. Our results may help establish an ultrafast experimental and theoretical methodology to extract the propagation dynamics of *L* currents.

## Conceptual background

Our approach follows the idea that *L* and *S* currents have the same symmetry properties yet potentially different dynamics on ultrashort time and length scales^1,8,9,14^. As depicted in Fig. 1, a femtosecond optical pump pulse excites an FM|PM stack and triggers ultrafast *S* and *L* currents with density *j*_*S*_ and *j*_*L*_, respectively, along the *z* axis from FM to PM. *SC*C and *LC*C result in ultrafast in-plane charge currents acting as sources of a terahertz electromagnetic pulse^28^. The resulting terahertz electric-field amplitude *E*(*t*) directly behind the sample is proportional to the sheet charge current *I*_*C*_(*t*), that is,

1 $$E\left(t\right)\propto {I}_{{{C}}}\left(t\right)={\int }_{\!\!\!-{d}_{{\rm{FM}}}}^{{d}_{{\rm{PM}}}}{\rm{d}}z\left[{{{\theta }}}_{{{{LC}}}}\left(z\right){j}_{L}\left(z,t\right)+{{{\theta }}}_{{{{SC}}}}\left(z\right){j}_{S}\left(z,t\right)\right].$$

Here, *d*_FM_ and *d*_PM_ denote the FM and PM thickness, respectively, and *θ*_*LC*_(*z*) and *θ*_*SC*_(*z*) describe the local efficiency of the instantaneous *LC*C and *SC*C, respectively. They include microscopic mechanisms like the ISHE or IOHE^27,29^, occurring in the bulk, or the ISREE and IOREE, which require regions of broken inversion symmetry such as interfaces^30,31^. Equation (1) neglects contributions due to magnetic-dipole radiation and contributions of photocurrents even in magnetization, because both can be discriminated experimentally^32,33^.

To understand the emergence of *j*_*S*_ and *j*_*L*_, we first note that sudden laser heating of the FM induces an *S* accumulation *μ*_*S*_. Here, *μ*_*S*_ is proportional to the excess *S* magnetization, that is, the difference between the instantaneous *S* magnetization and the equilibrium *S* magnetization that would be attained at the instantaneous elevated electron temperature^33–36^. Consequently, the FM releases *S* at a rate proportional to *μ*_*S*_, by transferring *S* to both the crystal lattice through, for example, spin flips, and to the PM by, for example, a spin-current density *j*_*S*_ ∝ *μ*_*S*_. Analogous to *S*, we expect that laser heating also induces an *L* accumulation *μ*_*L*_ and, in turn, *L* transport with *j*_*L*_ ∝ *μ*_*L*_.

Recent studies on single-element FMs like Ni showed that the *S*-type and *L*-type magnetizations exhibit very similar ultrafast dynamics following laser excitation^37–39^. Therefore, we expect *μ*_*L*_(*t*) ∝ *μ*_*S*_(*t*), where the ratio *μ*_*L*_/*μ*_*S*_ is FM-dependent^12^. Despite these identical driving dynamics, the resulting *j*_*L*_(*z*, *t*) and *j*_*S*_(*z*, *t*) (Fig. 1) can have very different evolutions because *S* and *L* may propagate differently through the FM/PM interface and the PM bulk.

## Experiment details

We study FM|PM thin-film stacks, where the two FMs Py and Ni are chosen for their high efficiency in generating *S* and *L* currents, respectively^9^. The chosen PMs have a strong ISHE (Pt, W) and IOHE (W, Ti) response. The reported ISHE signs are opposite for Pt versus W, with a vanishing ISHE in Ti. By contrast, the expected IOHE signs are the same for all three PMs^40^. The studied FM|PM thin-film stacks are deposited onto glass (thickness, 500 µm) or thermally oxidized Si substrates (thickness, 625 μm; Supplementary Fig. 1 and Methods). They are characterized by optical and terahertz transmission spectroscopy^41^, yielding the pump absorptance, d.c. conductivity and Drude relaxation rate (Supplementary Fig. 2).

In our experiment (Fig. 1), ultrashort laser pulses (10 fs nominal duration, 800 nm centre wavelength, 80 MHz repetition rate, 1.9 nJ pulse energy, 0.2 mJ cm^−2^ incident fluence) excite the sample. We record the emitted terahertz radiation by electro-optic sampling in a ZnTe(110) (thickness 1 mm or 10 µm) or GaP(110) (250 μm) crystal^42^. The resulting terahertz emission signal *S*_THz_(**M**, *t*) versus time *t* and sample magnetization **M** equals the terahertz electric-field waveform *E* (Fig. 1) convoluted with a set-up response function^43^. The presented data are smoothed by convolution with a Gaussian (80 fs full-width at half-maximum) for better visibility, unless noted otherwise.

All experiments are performed under ambient conditions unless stated otherwise. We apply an in-plane magnetic field of ~10 mT to the sample and monitor the terahertz field component perpendicular to **M**. The component parallel to **M** is found to be minor (Supplementary Fig. 3). Measurements with linearly and circularly polarized pump pulses reveal a negligible impact of the pump polarization on the terahertz emission (Supplementary Fig. 4). We checked explicitly that *S*_THz_(**M**, *t*) from the Ni-based samples increases almost linearly with pump fluence (Supplementary Fig. 5).

To isolate magnetic effects, we focus on the odd-in-**M** signal *S*_THz_(*t*) = [*S*_THz_(+**M**, *t*) − *S*_THz_(−**M**, *t*)]/2. The even-in-**M** signal components are more than two orders of magnitude smaller. Measurements on reversed samples reveal that *S*_THz_ predominantly arises from structural inversion asymmetry, consistent with Fig. 1. Minor contributions unrelated to structural inversion asymmetry most likely arise from magnetic-dipole radiation due to ultrafast demagnetization (Supplementary Fig. 6)^33^.

## Results

### Ferromagnetic Py versus Ni

**Fig. 2:**
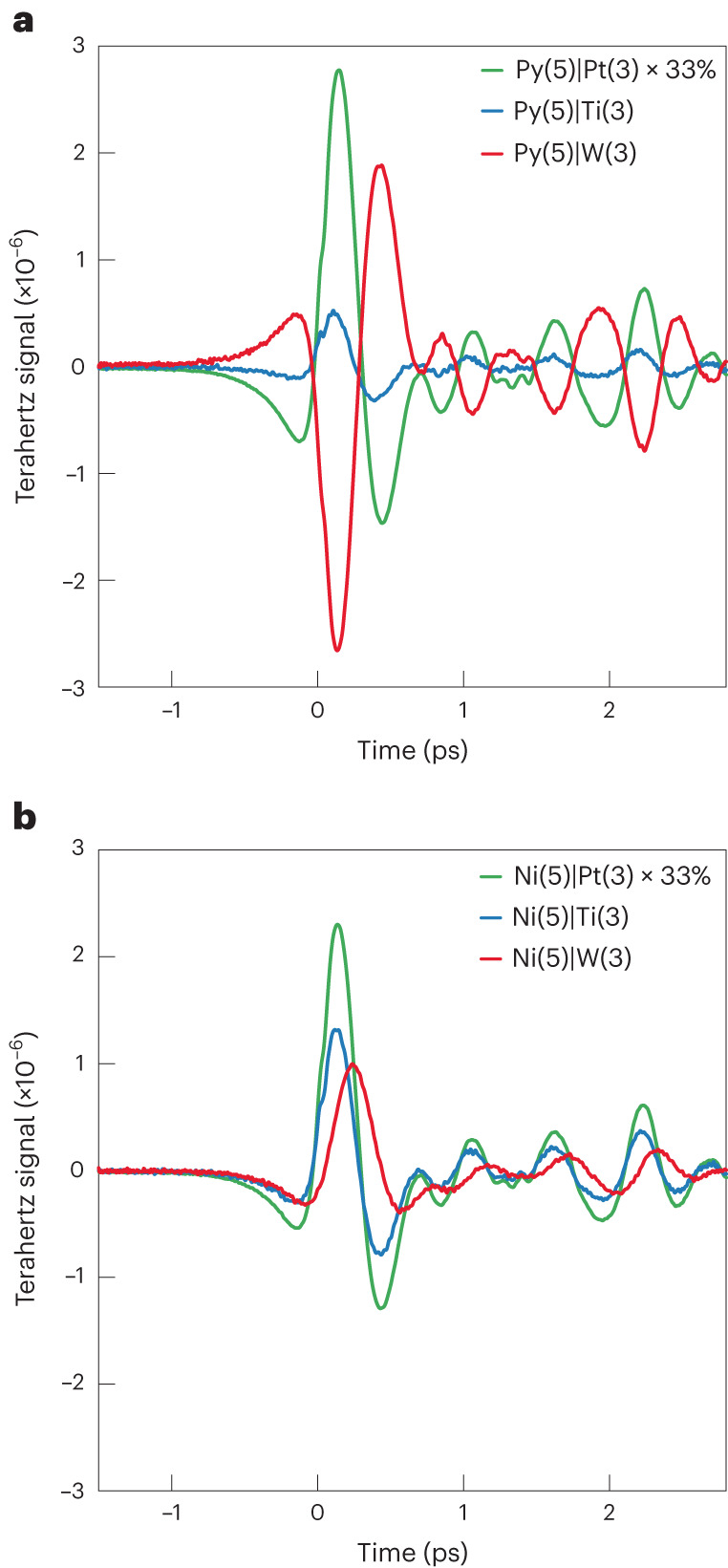
Terahertz raw data. **a**,**b**, Terahertz emission signals *S*_THz_(*t*) from FM|PM stacks with FM = Py (**a**) and FM = Ni (**b**) capped with PM = Pt, W or Ti. In both panels, the Pt-based sample signals are multiplied by 33%. Film thicknesses in nanometres are given as numerals in parentheses. As a terahertz detector, a ZnTe(110) crystal (thickness 1 mm) was used.

Figure 2a shows terahertz emission signals *S*_THz_ from Py|PM samples, where PM is Pt, W or Ti. All three waveforms have an identical shape. Minor shape differences of $S_{\mathrm{THz}}^{\mathrm{Py|Ti}}$ versus $S_{\mathrm{THz}}^{\mathrm{Py|Pt}}$ are attributed to contributions unrelated to structural inversion asymmetry as described above and in Supplementary Fig. 7.

The relative signal magnitudes and the opposite polarities for PM = Pt and W are consistent with previous reports of ISHE-dominated terahertz emission^28^. The polarity of the Py|Ti signal is the same as that of Py|Pt and consistent with calculations and measurements, which found the same sign of the ISHE and IOHE in Pt and Ti, respectively^13,27,40^. Even though Ti has a sizeable *LC*C efficiency, our data imply $S_{\mathrm{THz}}^{\mathrm{Py|Ti}} \ll S_{\mathrm{THz}}^{\mathrm{Py|Pt}}$. We ascribe this observation to a small *L*-current amplitude injected into Ti, consistent with the small *L* component of the Py magnetization^9^.

To summarize, for Py|PM, our terahertz signals are consistent with the transport of predominantly *S* and *L* into the PM bulk and its conversion into a charge current through the ISHE and IOHE, respectively. At the FM/PM interface, a possible Rashba-type or skew-scattering-type *LC*C or *SC*C^44^ may make an additional yet relatively small contribution.

When the FM of Py is replaced by Ni, the signal polarity remains identical for PM = Pt and Ti, and the two waveforms for the different FMs exhibit identical dynamics (Fig. 2b and Supplementary Fig. 8). By stark contrast, the signal polarity for Ni|W reverses with respect to Py|W, the waveform changes shape, and its maximum appears delayed compared to Py|W. This striking observation indicates that Py|W and Ni|W show different photocurrent mechanisms, the dominance of which depends sensitively on the FM. To understand the different dynamics in Ni|W, we next vary the W thickness.

### Impact of W thickness on current dynamics in Ni|W

**Fig. 3:**
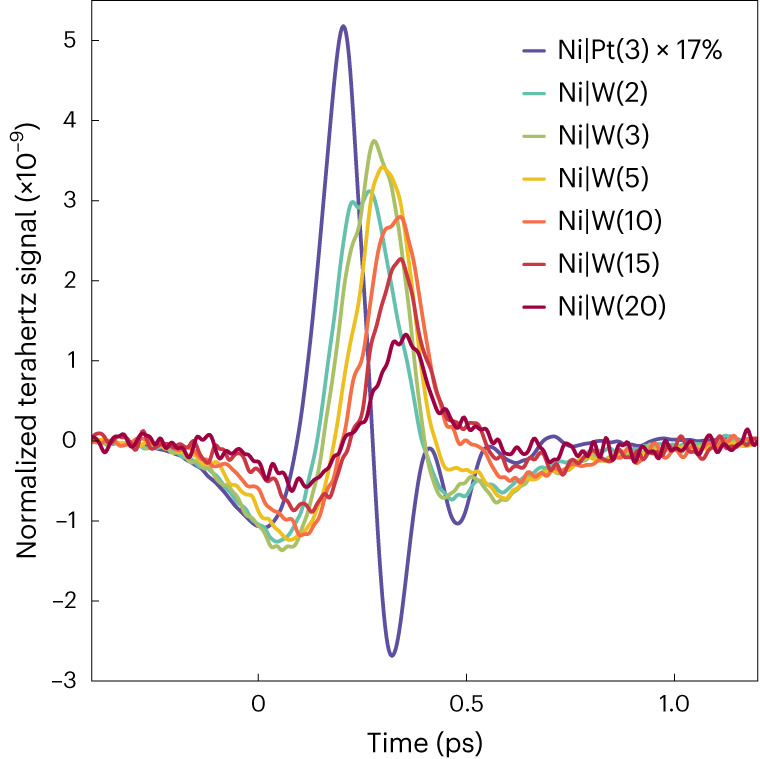
Impact of W thickness in Ni|W. Terahertz emission signals for Ni|W samples with varying W thickness, normalized to the absorbed pump-pulse fraction in the Ni layer and to the sample impedance (see Methods and Supplementary Table 1). Note the rescaling of the reference signal from Ni|Pt. Film thicknesses in nanometres are given as numerals in parentheses, except for Ni, which was always 5 nm thick. A GaP(110) crystal (thickness 250 µm) was used as a terahertz detector.

Figure 3 shows terahertz emission signals from Ni|W(*d*_W_) for various W thicknesses *d*_W_ and from a Ni|Pt reference sample. Consistent with Fig. 2b, we see a clear trend with increasing *d*_W_ relative to Ni|Pt: *S*_THz_(*t*) has the same sign, decreases with increasing *d*_W_ and reshapes considerably from being asymmetric (Ni|Pt) to being more symmetric (Ni|W) around the signal maximum. Interestingly, *d*_W_ = 2 nm already induces a shift of the signal maximum by about 100 fs compared to Ni|Pt.

Notably, the changes in terahertz signal dynamics solely originate from changing the PM thickness. Therefore, the FM is considered as a PM-independent *S* and *L* injector in the following.

To obtain a sample-intrinsic measurement of the *L* transport and conversion dynamics, we extract the sheet charge current *I*_*C*_(*t*) flowing in Ni|W (equation (1)) normalized to the absorbed pump-pulse fluence in Ni. This procedure eliminates any impact of sample exchange on the pump-pulse-absorption efficiency, sample impedance or set-up response function (Methods).

**Fig. 4:**
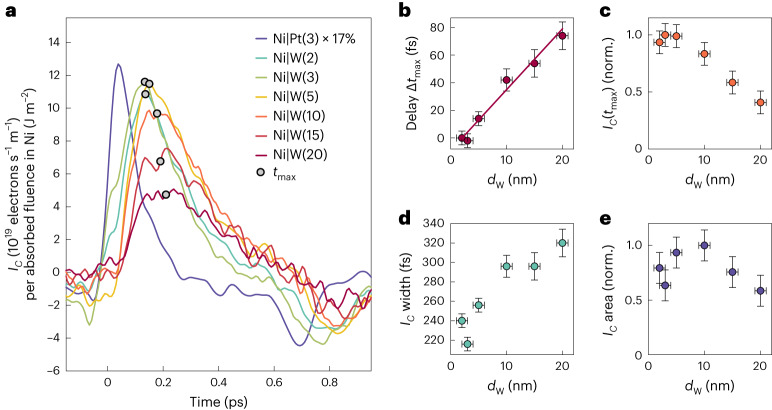
Ultrafast charge currents in Ni|W. **a**, Charge sheet currents *I*_*C*_(*t*) in Ni|W for various W thicknesses *d*_W_ as extracted from the data of Fig. 3. The feature at 0.8 ps is a remainder of a terahertz field reflection echo in the 10 µm ZnTe electro-optic detection crystal (Methods). Film thicknesses in nanometres are given as numerals in parentheses, except for Ni, which was always 5 nm thick. Note the rescaling of the Pt-based sample signal. The apparent signal maxima *I*_*C*_(*t*_max_) values are highlighted by circular markers. **b**, Extracted time delay Δ*t*_max_ relative to Ni(5 nm)|W(2 nm) with a straight line as a guide to the eye. **c**, Relative (normalized, norm.) amplitude of *I*_*C*_(*t*_max_). **d**, Full-width of *I*_C_(*t*) at half-maximum. **e**, Time-integrated *I*_*C*_(*t*) over 0.0–0.7 ps versus *d*_W_ from the data in **a**. Details of the error estimates are given in Methods.

Figure 4a displays *I*_*C*_(*t*) in Ni|W with a resolution of 50 fs for various W thicknesses *d*_W_. The *I*_*C*_(*t*) traces have striking features: (1) They exhibit the same polarity as Ni|Pt. (2) The *I*_*C*_ amplitude decreases approximately linearly with *d*_W_ to about 50% after 20 nm (Fig. 4c), indicating attenuation and dispersion upon propagation. (3) The *I*_*C*_ maximum shifts by delays Δ*t*_max_ ∝ *d*_W_ relative to *d*_W_ = 2 nm at a rate of Δ*t*_max_/*d*_W_ ≈ 4 fs nm^−1^ (Fig. 4b), implying a velocity of 0.25 nm fs^−1^. (4) The *I*_*C*_ width increases linearly at a rate of ≈8 fs nm^−1^ (Fig. 4d). (5) The time-integrated current ∫d*t I*_*C*_(*t*) only weakly decreases with *d*_W_, thereby indicating an extremely large relaxation length of >20 nm (Fig. 4e)^8,9,14^.

Feature (1) implies that *I*_*C*_(*t*) cannot arise from *S* transport. The reason is that the ISHE dominates the *SC*C in Pt and W, yet with opposite sign^28,45^. This conclusion is strongly supported by feature (2) because an *S* current in W would relax over distances of ≪20 nm (refs. ^41,46^). Our data, therefore, strongly indicate that *L* transport plus *LC*C dominates the terahertz charge current in Ni|W.

**Fig. 5:**
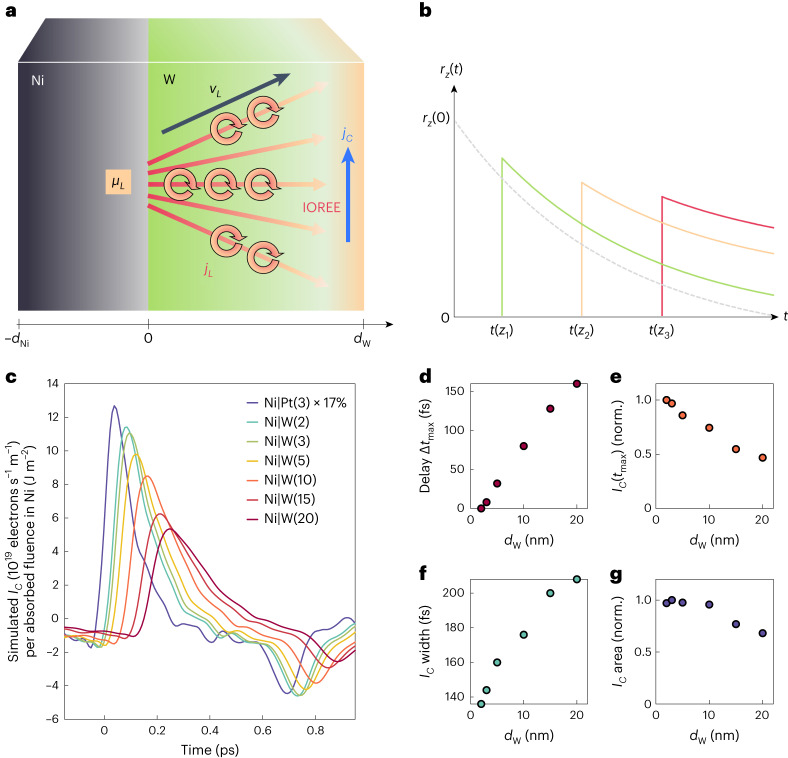
Model of *L* transport and IOREE in W. **a**, Schematic of the suggested scenario for *L* transport and *LC*C by the IOREE in Ni|W. The impulsive *L* accumulation *μ*_*L*_ launches *L* wave packets of various velocities **v**_*L*_ and, thus, an orbital current *j*_*L*_ into the W layer. Upon reaching the W back surface, *j*_*L*_ is converted into a transverse charge current *j*_*C*_ by the IOREE. In the experiment, the orbital currents of all point-like sources add up at the Ni/W interface**. b**, Qualitative ballistic current densities *r*_*z*_(*t*) in response to a fictitious δ(*t*)-like *μ*_*L*_ at different positions *z*_1_ < *z*_2_ < *z*_3_ in W. **c**, Simulated IOREE charge currents *I*_*C*_(*t*) obtained by equation (2) in the case of ballistic transport. Parameters are the *L* wave-packet velocity (|**v**_*L*_| = 0.14 fs nm^−1^; **a**), the *j*_*L*_ decay length (80 nm) and a global scaling factor. **d**, Extracted time delay Δ*t*_max_ relative to Ni(5 nm)|W(2 nm). **e**, Relative amplitude of *I*_*C*_(*t*_max_). **f**, Full-width of *I*_*C*_(*t*) at half-maximum. **g**, Time-integrated *I*_*C*_(*t*) over 0.0–0.7 ps versus *d*_W_ from the data in **c**.

Features (3) and (4) indicate a signal arising from ballistic-like transport of a pulse that is detected in an arrival layer. In this picture, the increasing width of *I*_*C*_(*t*) with *d*_W_ arises from a velocity dispersion along the *z* direction of the particles inside the pulse (Fig. 5a). Feature (5) implies a minor *LC*C in the W bulk because the integrated charge current ∫d*t I*_*C*_(*t*) would increase monotonically with *d*_W_ otherwise.

### Model of *L* current and IOREE in Ni|W

The preceding discussion suggests the following transport scenario in Ni|W. Excitation of the Ni layer induces a transient *S* and *L* accumulation (Fig. 1) with very similar dynamics as described previously^37–39^. The resulting *L* accumulation *μ*_*L*_(*t*) ∝ *μ*_*S*_(*t*) is monitored well by the ISHE charge current in Ni|Pt (Fig. 4a). It injects *L*-polarized electron wave packets into W, that is, coherent superpositions of Bloch states with non-zero net *L* (ref. ^1^; Fig. 5a). Finally, regions close to the W/SiO_2_ interface dominate the *LC*C. This interpretation is strongly supported by our ab initio calculations (Methods and Supplementary Figs. 9–11). The calculated *L* velocity (~0.1 nm fs^−1^; Supplementary Fig. 10) agrees well with our measurements (Fig. 4b). The calculated *LC*C (Supplementary Fig. 11) reveals a giant interfacial contribution (IOREE) in a thin W film with the same sign as the ISHE in Pt (Fig. 4a). An efficient interfacial *LC*C was already invoked previously^7,24,47–51^.

The suggested scenario explains all experimental charge-current features (1)–(5) (Fig. 4). As the *j*_*L*_ pulse propagates predominantly ballistically through W, its arrival in the W/SiO_2_ *LC*C region is delayed by a time Δ*t*_max_ ∝ *d*_W_. Likewise, *j*_*L*_ disperses due to different electron-velocity *z*-axis projections (Fig. 5a) and relaxes over a length of >20 nm.

To model the charge-current dynamics in Ni|W (Fig. 4), we assume *j*_*L*_ is solely driven by *μ*_*L*_ and, thus, given by the linear-response relationship (convolution)

2 $${j}_{L}\left(z,t\right)=\left({r}_{z} * {\mu}_{L}\right)\left(t\right)=\int_{-\infty}^{+\infty} {\rm{d}}\tau\ {r}_{z}\left(t-\tau \right){\mu}_{L}\left(\tau\right).$$

Here, *r*_*z*_ is the *L* current density at position *z* following a fictitious δ(*t*)-like *L* accumulation in Ni, and *μ*_*L*_(*τ*) ∝ *μ*_*S*_(*τ*) is given by the measured Ni|Pt charge current (Fig. 4a). Assuming an instantaneous IOREE response at *z* = *d*_W_, *I*_*C*_(*t*) is proportional to *j*_*L*_(*d*_W_, *t*).

In the Methods, we analytically calculate the response function *r*_*z*_ for the cases of ballistic transport (equations (9) and (10) and Fig. 5b) and diffusive transport (equation (12)). For ballistic transport, the modelled *I*_*C*_(*t*) curves (Fig. 5c) reproduce the measured Ni|W charge currents (Fig. 4a) semiquantitatively for a relaxation length of 80 nm and a dominant *L* wave-packet velocity of *v*_*L*_ = 0.14 nm fs^−1^. These values agree well with the estimates given previously (Fig. 4b–e) and with the calculated orbital velocity (Supplementary Fig. 10). We did not attempt to obtain even better agreement of the modelled and measured *I*_*C*_(*t*) by considering different distributions of the *L* velocity directions (Fig. 5a and equation (9)).

By contrast, for diffusive transport, our model reproduces the experimental data less favourably (Supplementary Fig. 12). Therefore, the observed *L* currents (Fig. 4) have a significant ballistic component.

To summarize, the terahertz charge currents in Ni|W (Fig. 4) arise from *L* currents injected into W. The charge-current generation (equation (1)) is dominated by *LC*C at the W/SiO_2_ interface. The extremely long-range *j*_*L*_ in W is a unique feature of orbitronic materials, first indications of which were found previously in Ti (refs. ^8,9^).

## Discussion

Our interpretation neglects other possible contributions to *I*_*C*_(*t*). First, the pump polarization independence rules out the inverse Faraday effect as a source of *S* (ref. ^52^) and *L* currents (Supplementary Fig. 4).

Second, for *j*_*S*_, a dominant Seebeck-type contribution from an electronic temperature difference Δ*T*_FM/PM_ across the Ni/PM interface is negligible^33^. For *j*_*L*_, we estimate Δ*T*_Ni/PM_ after pump-pulse absorption (Methods) and find Δ*T*_Ni/Pt_ = +400 K and Δ*T*_Ni/PM_ = −100 K for PM = Ti or W. The observed terahertz emission signals, by contrast, have the same sign for all three samples (Fig. 2b). Therefore, interfacial electronic temperature differences have a minor impact on *L* and *S* transport. Simulations further show that pump-intensity gradients in the FM and PM bulk are relatively small (Supplementary Fig. 13).

Third, angular-momentum transport in W by magnons is neglected because W is magnetically disordered. Acoustic phonons are excluded because the sound velocity in W is <0.01 nm fs^−1^ (ref. ^53^) and, thus, considerably below the observed transport velocity. An outstandingly long-range *S* propagation is ruled out because the Drude scattering times of all samples are ≪50 fs (Supplementary Fig. 2) and, thus, substantially shorter than the *I*_*C*_(*t*) peak delays (Fig. 4b).

Fourth, even though the IOREE dominates the charge-current generation in Ni|W, the positive shoulder-like feature at around time zero for *d*_W_ ≤ 3 nm in Fig. 4a may indicate a small contribution of bulk *LC*C, that is, the IOHE, consistent with the IOHE sign in W. An *L*-to-*S* conversion plus ISHE in the PM^23^ might contribute but is considered negligible here given the good agreement of the measurements (Fig. 4) and model (Fig. 5). The dominance of *j*_*L*_ in Ni|W highlights the role of Ni as an *L*-current source and indicates that the Ni/W interface may transmit *j*_*L*_ more efficiently than *j*_*S*_.

Interestingly, a detailed comparison of Fig. 2a,b reveals pronounced amplitude changes for PM = W or Pt when changing the FM from Py to Ni. These differences are related to the intricate interplay of all parameters in equation (1), in addition to relative changes of *μ*_*S*_ and *μ*_*L*_ and of interface transmission coefficients for *j*_*S*_ and *j*_*L*_.

We emphasize that samples deposited on Si rather than glass substrates show very similar terahertz emission characteristics (Supplementary Fig. 1), demonstrating the robustness of the observed effects. When adding a Cu layer on top of the Ni|W sample, we find similar terahertz emission signals (Supplementary Fig. 14). Interestingly, a Cu interlayer in Ni|Cu|W only slightly modifies the terahertz signal, suggesting that Cu does not block *L* transport strongly.

Regarding earlier reports of different terahertz emission dynamics in Fe|Au and Fe|Ru samples^29^, we note that a possible IOREE in Ru seems possible in hindsight. This interpretation might explain the seemingly strong dependence of the Fe|Ru terahertz emission dynamics on growth details^29,54,55^.

## Conclusions

We observe terahertz emission signals from optically excited Ni|W stacks that are consistently assigned to the ultrafast injection of *L* currents into W and long-distance ballistic transport through W. Remarkably, we find strong indications for a dominant IOREE in both experiment and theory. This result can be considered as time-domain signature of the long-range nature of orbital currents and the IOREE in W.

Our study highlights the power of broadband terahertz emission spectroscopy in disentangling *S* and *L* transport as well as Hall-like and Rashba–Edelstein-like conversion processes based on their dynamics. We find that Py versus Ni are, respectively, attractive *S* and *L* sources, whereas Pt versus W are, respectively, good *S*-to-charge and *L*-to-charge converters with distinctly different efficiency and dynamics for *S* versus *L*. We believe that our results are a significant step toward the identification of ideal sources and detectors of either *S* or *L* currents, which will strongly benefit from accurate theoretical predictions.

## Methods

### Current extraction

To extract the in-plane sheet current *I*_*C*_ flowing inside the sample from the measured terahertz signal *S*_THz_, we first measure our set-up response function *H*_*SE*_ by having a reference electro-optic emitter (50 μm GaP on a 500 μm glass substrate) at the same position as the sample, which yields a reference terahertz signal $S_{\mathrm{THz}}^{\mathrm{ref}}$. By calculating the emitted terahertz electric field *E*^ref^ from that reference emitter, *H*_*SE*_ is determined by solving the convolution ${{{S}}}_{{\rm{THz}}}^{{\rm{ref}}}\left({{t}}\right)=\left({{{H}}}_{{{SE}}} * {{{E}}}^{{\rm{ref}}}\right)\left({{t}}\right)$ (equation (2)) for *H*_*SE*_ (ref. ^43^). Further measured inputs for this calculation are the excitation spot size with a full-width at half-maximum of 22 μm, the excitation pulse energy of 1.9 nJ and a Fourier-transform-limited pump pulse with a spectrum centred at 800 nm and a full-width at half-maximum of 110 nm. We perform the deconvolution directly in the time domain by recasting it as a matrix equation^16^.

Next, the electric field *E* directly behind the sample is obtained from the recorded terahertz signal *S*_THz_ with the help of the derived function *H*_*SE*_ by solving the analogous equation *S*_THz_(*t*) = (*H*_*SE*_ * *E*)(*t*) for *E*. Finally, the sheet charge current (Supplementary Table 1) as shown in Fig. 3 is derived from a generalized Ohm’s law^28^, which in the frequency domain at frequency *ω*/2π reads

3 $$E\left(\omega \right)={eZ}\left(\omega \right){I}_{{{C}}}\left(\omega \right).$$

Here, −*e* is the electron charge and the sample impedance *Z*(*ω*) is given by *Z*_0_/[1 + *n*_sub_ + *Z*_0_*dσ*(*ω*)] with the free-space impedance *Z*_0_, the substrate refractive index *n*_sub_ ≈ 2 (ref. ^56^) and the metal-stack thickness *d* = *d*_FM_ + *d*_PM_. The measured mean sample conductivity *σ* (Supplementary Table 1) is approximately frequency-independent due to the large Drude scattering rate (Supplementary Fig. 2). To enable a comparison of terahertz currents from different samples, we normalize *I*_*C*_ by the absorbed fluence in the FM. The data shown in Fig. 3 were obtained in a dry-air atmosphere.

### Sample preparation

The FM|PM samples (FM = Ni and Py, PM = Pt, Ti, Cu and W) are fabricated on glass substrates (thickness 500 μm) or thermally oxidized Si substrates (625 μm) by radio-frequency magnetron sputtering under an Ar atmosphere of 6N purity. The sample structure and thickness are described in Supplementary Table 1. For the sputtering, the base pressure in the chamber is lower than 5 × 10^−7^ Pa. To avoid oxidation, SiO_2_ (thickness 4 nm) is sputtered on the surface of the films. All sputtering processes are performed at room temperature. The W films are predominantly in the β-phase for *d*_W_ < 10 nm, with an α-phase content that grows with *d*_W_ and dominates for *d*_W_ > 10 nm (ref. ^9^).

### Estimate of electronic temperatures

We calculate the electronic temperature increase Δ*T*_e0_ upon pump-pulse absorption by

4 $$\Delta {T}_{{\rm{e}}0}=\sqrt{{T}_{0}^{2}+\frac{2{F}_{l}}{\gamma d}}-{T}_{0}.$$

Here, *T*_0_ = 300 K is the ambient temperature, *F*_*l*_ is the absorbed fluence in the respective layer *l* = FM or PM (Supplementary Table 1), *d* is the layer thickness and *γT*_e_ is the specific electronic heat capacity at electronic temperature *T*_e_ with *γ* = 300 J m^−3^ K^−2^ for W, 320 J m^−3^ K^−2^ for Ni, 330 J m^−3^ K^−2^ for Ti and 90 J m^−3^ K^−2^ for Pt (ref. ^57^).

To obtain the absorbed fluences in each layer, we note that the pump electric field is almost constant throughout the sample (Supplementary Fig. 9). Therefore, the local pump absorption scales solely with the imaginary part Im*ε* of the dielectric function *ε* at a wavelength of 800 nm, which equals 22.07 for Ni, 9.31 for Pt, 19.41 for Ti and 19.71 for W (ref. ^58^). Consequently, the absorbed fluence is determined by

5 $${F}_{l}={F}_{{\rm{tot}}}\frac{{d}_{l}{\rm{Im}}{\varepsilon }_{l}}{{d}_{{\rm{FM}}}{\rm{Im}}{\varepsilon }_{{\rm{FM}}}+{d}_{{\rm{PM}}}{\rm{Im}}{\varepsilon }_{{\rm{PM}}}}$$

with the total absorbed fluence *F*_tot_ that is obtained from the absorbed pump power (Supplementary Table 1) and the beam size on the sample (as described previously).

### Experimental error estimation

The error bars for the maximum position *t*_max_ of the transient charge current *I*_*C*_(*t*) (Fig. 4b) are estimated as ±20% of the read-off delay value (circular markers in Fig. 4a), but no less than 5 fs. The uncertainty in the relative amplitude of *I*_*C*_(*t*_max_) (Fig. 4c) and the relative area of *I*_*C*_(*t*) (Fig. 4e) is, respectively, estimated as ±20% and ±10%, both reflecting the typical signal-to-noise ratio of the extracted current traces (Fig. 4a). The error bars for the full-width of *I*_*C*_(*t*) at half-maximum (Fig. 4d) are obtained from the uncertainty of the delay (Fig. 4b) with subsequent multiplication by $\sqrt{2}$, which accounts for the error propagation of a difference of two quantities.

### Model of *L* transport

To model the ballistic current in the PM, we assume that a δ(*t*)-like transient *L* accumulation in the FM generates an electronic wave packet, which has orbital angular momentum Δ*L*_**k**0_ along the direction of the FM magnetization **M** and mean wave vector **k** in the PM right behind the FM/PM interface, that is, at *z* = 0^+^ (Fig. 5a).

In the case of purely ballistic transport, this wave packet propagates into the PM bulk according to Δ*L*_**k**_(*z*, *t*) = Δ*L*_**k**0_ δ(*z* − *v*_**k***z*_*t*), where *v*_**k***z*_ is the *z* component of the wave-packet group velocity. Note that we restrict ourselves to **k** values with non-negative *v*_**k***z*_ values. The total pump-induced *L* current density flowing into the depth of the PM is for *z* > 0 given by the sum

6 $${r}_{z}\left(t\right)=\mathop{\sum }\limits_{{\bf{k}},{v}_{{\bf{k}}z}\ge 0}\Delta {L}_{{\bf{k}}0}{v}_{{\bf{k}}z}\updelta\left(z-{v}_{{\bf{k}}z}t\right).$$

Assuming that Δ*L*_**k**0_ arises from states not too far from the Fermi energy, the summation of equation (6) is approximately proportional to an integration over the Fermi surface parts with *v*_**k***z*_ ≥ 0. One obtains

7 $${r}_{z}(t)={\mathrm{{e}}}^{-t/\tau }{\int }_{0}^{\infty }{\rm{d}}{v}_{z}w({v}_{z}){v}_{z}\updelta (z-{v}_{z}t)$$

where *z* > 0, and

8 $$w({v}_{z})=\sum _{{\bf{k}},{v}_{{\bf{k}}z}\ge 0}\Delta {L}_{{\bf{k}}0}\updelta ({v}_{{\bf{k}}z}-{v}_{z})$$

is the *L* weight of the *z*-axis group velocity *v*_*z*_. In equation (7), we phenomenologically account for the relaxation of the ballistic current with time constant *τ* by introducing the factor e^−*t*/*τ*^. Performing the integration of equation (7) yields

9 $${r}_{z}\left(t\right)=\frac{{{\rm{e}}}^{-t/\tau }}{t}\frac{z}{t}w\left(\frac{z}{t}\right).$$

To determine a plausible shape of *w*(*v*), we note that Δ*L*_**k**0_ is non-zero within several 0.1 eV around the Fermi energy *E*_F_ owing to the width of the photoexcited and rapidly relaxing electron distribution^33^. Assuming a spherical Fermi surface and isotropic Δ*L*_**k**0_, we have Δ*L*_**k**0_ ∝ δ(*E*_**k**_ − *E*_F_) and *v*_**k***z*_ = *v*_F_cos*θ*, where *E*_**k**_ is the band structure, *v*_F_ is the Fermi velocity and *θ* is the angle between **k** and the *z* axis. Therefore, after turning equation (8) into an integral, the integrand $\Delta {L}_{{\bf{k}}0}\updelta\left({v}_{{\rm{F}}}{{\cos }}\theta -{v}_{z}\right)$ ${{\rm{d}}^{3}{\bf{k}}}$ becomes proportional to $\updelta\left({v}_{{\rm{F}}}{{\cos }}\theta -{v}_{z}\right)$ ${\rm{d}}{{\cos }}\theta$ in spherical coordinates, leading to

10 $$w\left({v}_{z}\right)\propto {\int }_{0}^{1}{\rm{d}}{{\cos }}\theta\ \updelta\left({v}_{{\rm{F}}}{{\cos }}\theta -{v}_{z}\right)\propto \varTheta \left({v}_{{\rm{F}}}-{v}_{z}\right),$$

where Θ is the Heaviside step function. In other words, all velocities ${v}_{z}={v}_{{\rm{F}}}{{\cos }}\theta$ from 0 to *v*_F_ have equal weight.

In the case of purely diffusive transport, we use the *L* diffusion equation for *μ*_*L*_ (ref. ^23^). With a localized accumulation *μ*_*L*_(*z*, *t*) ∝ δ(*z*) at time *t* ≈ 0, the accumulation disperses according to the well-known solution

11 $${\mu }_{L}\left(z,t\right)\propto \frac{1}{\sqrt{Dt}}{{\exp }}\left(-\frac{{z}^{2}}{4Dt}\right)$$

for *z* > 0 and *t* > 0. Here, *D* is the diffusion coefficient that equals ${v}_{L}^{2}\tau /3$ in the case of a spherical **k**-space surface carrying the *L* wave packets, *v*_*L*_ is their mean group velocity, and *τ* is their velocity relaxation time. To determine the current density, we apply Fick’s law^23^ *j*_*L*_ = −*D*∂*μ*_*L*_/∂*z* to equation (11) and obtain

12 $${r}_{z}\left(t\right)\propto \varTheta \left(t\right)\frac{z}{t}\frac{1}{\sqrt{Dt}}{{\exp }}\left(-\frac{{z}^{2}}{4Dt}\right).$$

### Ab initio estimate of the *L* velocity

The orbital velocity is estimated for the bulk W in a body-centred cubic structure. The ab initio self-consistent calculation of the electronic states is performed within density functional theory by using the FLEUR code^2^, which implements the full-potential linearly augmented plane-wave (FLAPW) method^59^. The exchange-correlation effect is included in the scheme of the generalized gradient approximation by using the Perdew–Burke–Ernzerhof functional^60^. The lattice parameter of the cubic unit cell is set to 5.96*a*_0_, where *a*_0_ is the Bohr radius. For the muffin-tin potential, we set *R*_MT_ = 2.5*a*_0_ for the radius and *l*_max_ = 12 for the maximum of the harmonic expansion. Further, we set the plane-wave cut-offs for the interstitial region to $4.0{a}_{0}^{-1}$, $10.1{a}_{0}^{-1}$ and $12.2{a}_{0}^{-1}$ for the basis set, the exchange-correlation functional and the charge density, respectively. For the **k**-points, a 16 × 16 × 16 Monkhorst–Pack mesh is defined.

From the converged electronic structure, we obtain the maximally localized Wannier functions by using the WANNIER90 code^61^. We use 18 Wannier states with $s,{p}_{x},{p}_{y},{p}_{z},{d}_{{z}^{2}},{d}_{{x}^{2}-{y}^{2}},{d}_{xy},{d}_{yz},{d}_{zx}$ symmetries for spin up and down as the initial guess. The maximum of the inner (frozen) energy window is set 5 eV above the Fermi energy for the disentanglement, and the outer energy window is defined by the minimum and maximum energies of the 36 valence states obtained from the FLAPW calculation. The Hamiltonian and the position, orbital-angular-momentum and spin-angular-momentum operators are transformed from the FLAPW basis into the maximally localized Wannier function basis. The resulting electronic band structure and texture of the orbital-angular-momentum operator **L** are displayed in Supplementary Fig. 9.

From this realistic tight-binding model, the orbital-momentum-weighted velocity averaged over the Fermi surface (FS) is calculated by

13 $${\langle {v}_{\alpha }{L}_{\beta }\rangle }_{{\rm{FS}}}=\frac{{\sum }_{n{\bf{k}}}{\,f}_{n{\bf{k}}}^{\,{\prime} }\langle n{\bf{k}}|({v}_{\alpha }{L}_{\beta }+{L}_{\beta }{v}_{\alpha })/2|n{\bf{k}}\rangle }{{\sum }_{n{\bf{k}}}{\,f}_{n{\bf{k}}}^{\,{\prime} }},$$

where *v*_*α*_ and *L*_*β*_ are the *α* component of the velocity and the *β* component of the orbital-angular-momentum operators, respectively. The |*n***k〉** is the eigenstate of the Hamiltonian with energy *E*_*n*__**k**_ and band index *n*, and ${f}_{n{\bf{k}}}^{\,{\prime} }$ is the energy derivative of the Fermi–Dirac distribution function. To polarize *L*_*β*_, we add a small orbital Zeeman coupling along the *β* direction to the bare Hamiltonian. We confirm that the result of equation (13) changes by less than 1% when the orbital Zeeman splitting is increased from 10 meV to 30 meV. The **k**-space integrals in equation (13) are performed on a 256 × 256 × 256 mesh.

The orbital velocity is estimated by

14 $${\left\langle {v}_{\alpha }^{{L}_{\beta }}\right\rangle }_{{\rm{FS}}}=\frac{{\left\langle {v}_{\alpha }{L}_{\beta }\right\rangle }_{{\rm{FS}}}}{\sqrt{{\left\langle {L}_{\beta }^{2}\right\rangle }_{{\rm{FS}}}}},$$

where ${\langle {L}_{\beta }^{2}\rangle }_{{\rm{FS}}}$ is obtained by equation (13), but with *v*_*α*_ replaced by *L*_*β*_. The result is shown in Supplementary Fig. 10.

### Details of ab initio *LC*C calculations

A thin W stack of 19 body-centred cubic (110) atomic layers is calculated by the self-consistent ab initio method using the same FLAPW parameters as for the bulk calculation given previously, except for the mesh of **k**-points for which we use a 24 × 24 Monkhorst–Pack mesh. For Wannierization, we obtain 342 maximally localized Wannier functions, starting from Wannier states with $s,{p}_{x},{p}_{y},{p}_{z},{d}_{{z}^{2}},{d}_{{x}^{2}-{y}^{2}},{d}_{xy},{d}_{yz},{d}_{zx}$ symmetries for spin up and down as the initial guess. We define the maximum of the inner window at 2 eV above the Fermi energy.

From the Hamiltonian of the W thin film, the **k**-space orbital-angular-momentum texture at the Fermi surface is obtained by

15 $${\langle {{\bf{L}}}_{{\rm{top}}}\rangle }_{{\rm{FS}}}({\bf{k}})=-4{k}_{{\rm{B}}}T\sum _{n}{f}_{n{\bf{k}}}^{\,{\prime} }{\langle {{\bf{L}}}_{{\rm{top}}}\rangle }_{n{\bf{k}}},$$

where ${\langle {{\bf{L}}}_{{\rm{top}}}\rangle }_{n{\bf{k}}}=\langle n{\bf{k}}|{{\bf{L}}}_{{\rm{top}}}|n{\bf{k}}\rangle$ is the expectation value of the orbital angular momentum for the two atoms on the top surface, *T* = 300 K is temperature, and *k*_B_ is the Boltzmann constant.

To calculate the charge current due to *LC*C, we consider the orbital-dependent chemical potential

16 $$\frac{{\varepsilon }_{\beta \gamma }}{2}({r}_{\beta }{L}_{\gamma }+{L}_{\gamma }{r}_{\beta })$$

as a perturbation. Here, *L*_*γ*_ is the *γ* component of the orbital-angular-momentum operator, and *r*_*β*_ is the *β* component of the position operator, which is well-defined along the *z* axis (Fig. 1), and ${\varepsilon }_{\beta \gamma }$ can be interpreted as an orbital-dependent electric field. The charge-current density along the *α* direction is given by the Kubo formula

17 $$\langle {\,j}_{\alpha }\rangle =-\frac{e}{V}\sum _{{\bf{k}}nn^{\prime} }({\,f}_{n{\boldsymbol{k}}}-{f}_{n^{\prime} {\bf{k}}}){\rm{Re}}\frac{\langle n{\bf{k}}|{v}_{\alpha }|n^{\prime} {\bf{k}}\rangle \langle n^{\prime} {\bf{k}}|V|n^{\prime} {\bf{k}}\rangle }{{E}_{n{\bf{k}}}-{E}_{n^{\prime} {\bf{k}}}+{\rm{i}}\varGamma }$$

where *V* is the volume of the system and *Γ* = 25 meV is a phenomenological broadening parameter. The *LC*C response is characterized by the tensor

18 $${\sigma }_{{{{LC}}}{\rm{C}},\alpha \beta }^{{L}_{\gamma }}=-\frac{e}{2V}\mathop{\sum }\limits_{{\bf{k}}n{n}^{{\prime} }}\left({\,f}_{n{\bf{k}}}-{f}_{{n}^{{\prime} }{\bf{k}}}\right){\rm{Re}}\frac{\left\langle n{\bf{k}},|,{v}_{\alpha },|,{n}^{{\prime} }{\bf{k}}\right\rangle \left\langle {n}^{{\prime} }{\bf{k}},|,\left({r}_{\beta }{L}_{\gamma }+{L}_{\gamma }{r}_{\beta }\right),|,{n}^{{\prime} }{\bf{k}}\right\rangle }{{E}_{n{\bf{k}}}-{E}_{{n}^{{\prime} }{\bf{k}}}+{\rm{i}}\varGamma },$$

which relates $\langle {j}_{a}\rangle$ and *ε*_*βγ*_ by $\langle {j}_{\alpha }\rangle ={\sigma }_{{LC\mathrm{C}},\alpha \beta }^{{L}_{\gamma }} \ \epsilon_{\beta \gamma}$. The **k**-space integral is performed on a 400 ×  400 mesh. The *z*-resolved *LC*C response is shown in Supplementary Fig. 11.

### Reporting summary

Further information on research design is available in the Nature Portfolio Reporting Summary linked to this article.

## Online content

Any methods, additional references, Nature Portfolio reporting summaries, source data, extended data, supplementary information, acknowledgements, peer review information; details of author contributions and competing interests; and statements of data and code availability are available at 10.1038/s41565-023-01470-8.

### Supplementary information

Supplementary Information Supplementary Figs. 1–14 and Table 1.

Reporting Summary

## Data Availability

The data that support the plots in the main text of this paper are openly available in Zenodo at 10.5281/zenodo.8020863. The data that support other findings of this study are available from the corresponding author on request.

## Code availability

Custom computer codes or algorithms used to generate results that are reported in the paper and central to its main claims are available from the corresponding author on request.
