# Publisher Correction: Time-domain observation of ballistic orbital-angular-momentum currents with giant relaxation length in tungsten

**DOI:** 10.1038/s41565-023-01504-1

**Published:** 2023-08-21

**Authors:** Tom S. Seifert, Dongwook Go, Hiroki Hayashi, Reza Rouzegar, Frank Freimuth, Kazuya Ando, Yuriy Mokrousov, Tobias Kampfrath

**Affiliations:** 1 Department of Physics, Freie Universität Berlin, Berlin, Germany; 2 Department of Physical Chemistry, Fritz-Haber-Institut der Max-Planck-Gesellschaft, Berlin, Germany; 3 Peter-Grünberg-Institut, Forschungszentrum Jülich, Jülich, Germany; 4 Institute of Physics, Johannes-Gutenberg-Universität Mainz, Mainz, Germany; 5 Department of Applied Physics and Physico-Informatics, Keio University, Yokohama, Japan; 6 Keio Institute of Pure and Applied Sciences, Keio University, Yokohama, Japan; 7 Center for Spintronics Research Network, Keio University, Yokohama, Japan

**Keywords:** Magnetic properties and materials, Spintronics, Terahertz optics

Correction to: *Nature Nanotechnology* 10.1038/s41565-023-01470-8. Published online 7 August 2023.

In the version of this article initially published, several edits made during production were missing from the published article. The labels “FM” and “PM” were missing from the grey and green sections of Fig. 1, respectively, and “*d*_FM_” and “*d*_Pm_” appeared in the coloured sections rather than at ends of the *z* axis. In the last paragraph of the introduction, in the text reading “The bandwidth and amplitude of the underlying burst of charge current decreases with W thickness, whereas its delay and width increase linearly,” reference to “width” was missing. In the last paragraph of “Conceptual background,” in the text reading “Therefore, we expect *μL*(*t*) ∝ *μS*(*t*), where the ratio *μL*/*μS* is FM-dependent,” the words “the ratio” were missing. In the third paragraph of the section “Impact of W thickness on current dynamics in Ni|W”, in the sentence reading “To obtain a sample-intrinsic measurement of the *L* transport and conversion dynamics, we extract the sheet charge current *I*_*C*_(*t*) flowing in Ni|W (equation (1)) normalized to the absorbed pump-pulse fluence in Ni,” “pump-pulse” replaces the original word “laser.” In the third paragraph of the section “Model of *L* current and IOREE in Ni|W,” in the text reading “To model the charge-current dynamics in Ni|W (Fig. 4),” the callout originally cited Fig. 3. In the text immediately following equation (11), now reading, in part, “Here, *D* is the diffusion coefficient *… v*_*L*_ is their mean group velocity,” “mean” was missing. Other minor formatting changes have also been made in the HTML and PDF versions of the article.

