# Supplementary material for: Time-domain observation of ballistic orbital-angular-momentum currents with giant relaxation length in tungsten
Source: Nat Nanotechnol. 2023 Aug 7;18(10):1132–8. doi: 10.1038/s41565-023-01470-8 (PMC10575790; doi:10.1038/s41565-023-01470-8)
Supplement: Supplementary file 1 — Supplementary Figs. 1–14 and Table 1. [file 41565_2023_1470_MOESM1_ESM.pdf]

nature nanotechnology

Article https://doi.org/10.1038/s41565-023-01470-8

Time-domain observation of ballistic orbital-angular-momentum currents with giant relaxation length in tungsten

In the format provided by the authors and unedited

Supplementary Information

Before showing the corresponding data, we summarize briefly the content of the Supplementary Materials:

- Samples on Si show qualitatively the same THz emission waveforms for Ni with Pt, W and Ti. Most importantly, the strong change in W dynamics is also observed on Si (Fig. S1). However, the THz waveforms of Si vs glass differ in the details, which might be related to slightly changed transport times.
- Drude scattering times are estimated to be $\ll 50$ fs for all studied samples (Figs. S2). None of the samples show any indication of a drastically different Drude scattering time compared to all other samples.
- Emitted THz signals are found to be linearly polarized and perpendicular to the sample magnetization (Fig. S3).
- Pump-polarization dependent studies (pump helicity and linear polarization direction) show a minor impact on the measured THz emission signal (Figs. S4).
- Ni|Ti and Ni|W samples, all fluence dependencies are to a good approximation linear with minor sublinearities (Fig. S5). Likewise, only minor changes in the THz waveform dynamics can be observed for different pumping fluences (Fig. S5).
- We perform THz emission measurements upon reversing the sample. Only the pure Ni film shows a dominant contribution even in sample reversal, which we ascribe to SIA or magnetic-dipole radiation (Fig. S6) [1, 2].
- For all Py-based bilayer samples, we find almost identical THz emission waveform shapes even for elevated PM thicknesses of 20 nm (Fig. S7).
- For Ni|Ti samples, we find almost identical THz emission dynamics to Ni|Pt (Fig. S8).
- *Ab-initio* calculations of the orbital polarization close to the W-layer surface, the estimated velocity of the orbital current and the interface concentrated LCC in a W film are shown in Figs. S9-S11.
- A comparison of purely ballistic vs purely diffusive motion for orbitally polarized wavepackets is shown in Fig. S12.
- Currents driven by pump-intensity gradients in thick films of Ni|W and Ni|Ti can be neglected (Fig. S13).
- Cu has only a minor impact on the emitted THz waveforms (Fig. S14), either as a spacer layer or as a capping layer, as confirmed by comparison to the same sample without Cu.
- All data in the Supplementary Materials was measured with a ZnTe(110) detection crystal (thickness 1 mm).

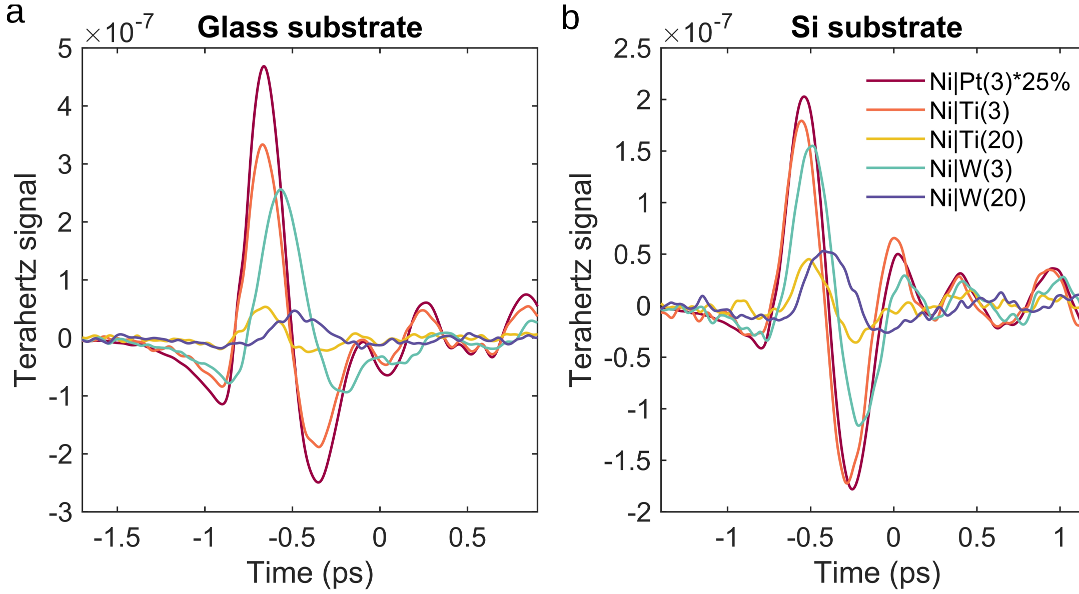

FIGURE S1: Si vs glass substrate. a, THz-emission waveforms from Ni|PM stacks on Si substrates. THz waveforms for Si based samples are multiplied by −1 to account for the reversed sample orientation due to the intransparency of the Si substrate for the pump pulse. **b**, THz-emission waveforms from Ni|PM stacks on glass substrates. Film thicknesses in nanometers are given as numerals in parenthesis, except for Ni layers, which are always 5 nm thick. Note the rescaling of the Ni|Pt sample THz waveforms.

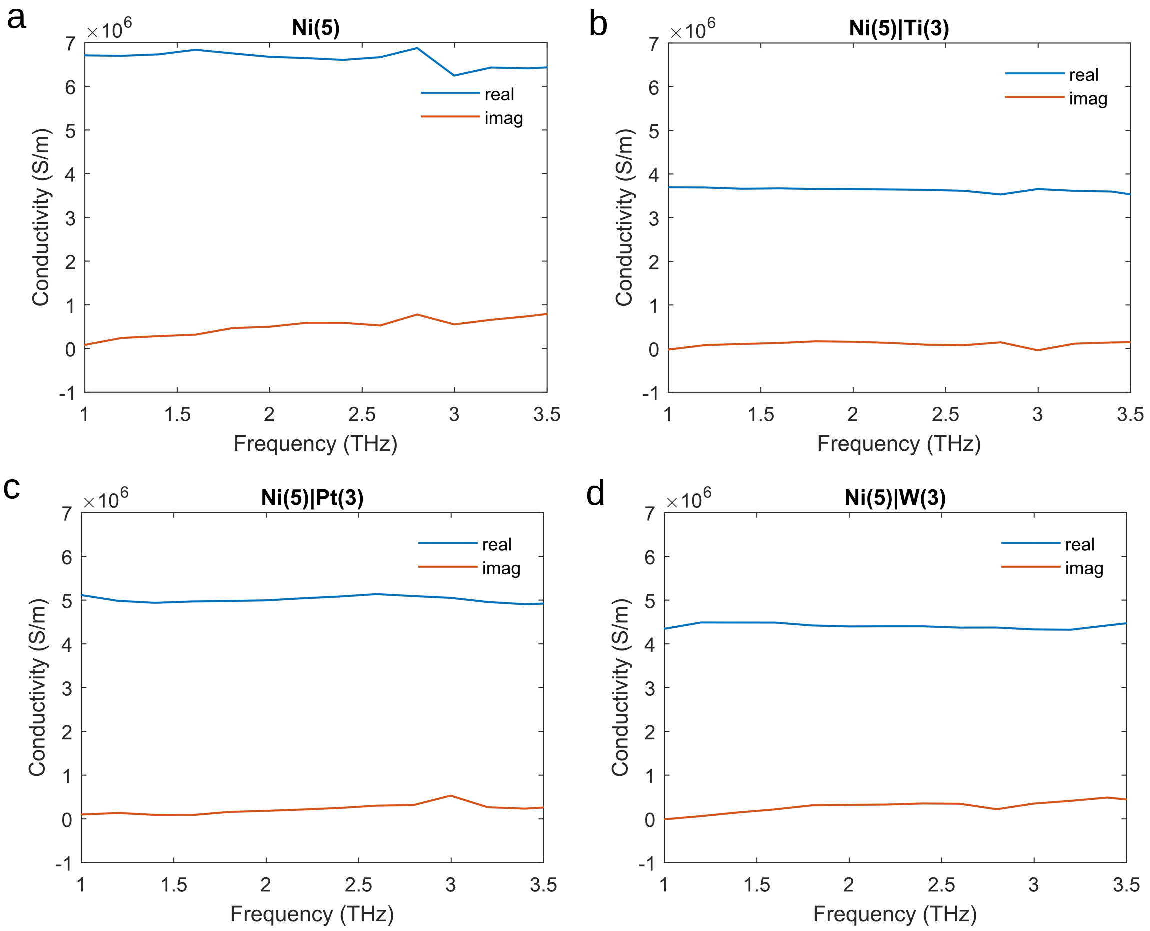

FIGURE S2: THz conductivities for samples on glass. Mean complex-valued THz conductivities obtained from THz-transmission measurements for **a**, Ni, **b**, Ni|Ti, **c**, Ni|Pt and **d**, Ni|W samples. The analysis is based on a thin-film formula [3] and assumes a THz refractive index of 2.1 for glass. Film thicknesses in nanometers are given as numerals in parentheses. In all panels, the extrapolated real (blue solid line) and imaginary parts (red) of the conductivity are expected to cross at frequencies $\Gamma/2\pi \gg 3$ THz. For a Drude-like conductivity, it follows that the current relaxation time $1/\Gamma$ is $\ll 50$ fs [4].

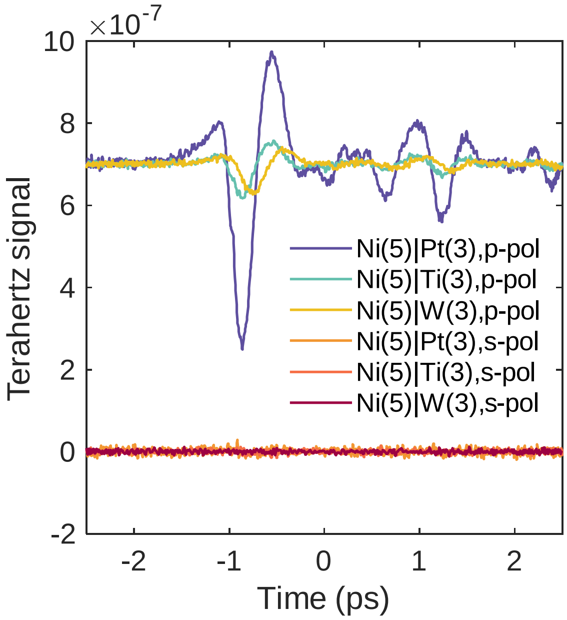

FIGURE S3: Polarization state of the THz-emission signal. Samples are magnetized along the s-direction, and pump pulses are polarized along the p-direction. Film thicknesses in nanometers are given as numerals in parenthesis. The vertically offset upper curves show the p-polarized THz-emission signal, whereas the lower curves show the s-polarized THz emission signal. This figure implies that the THz-emission signal is primarily polarized linearly and perpendicular to the sample magnetization.

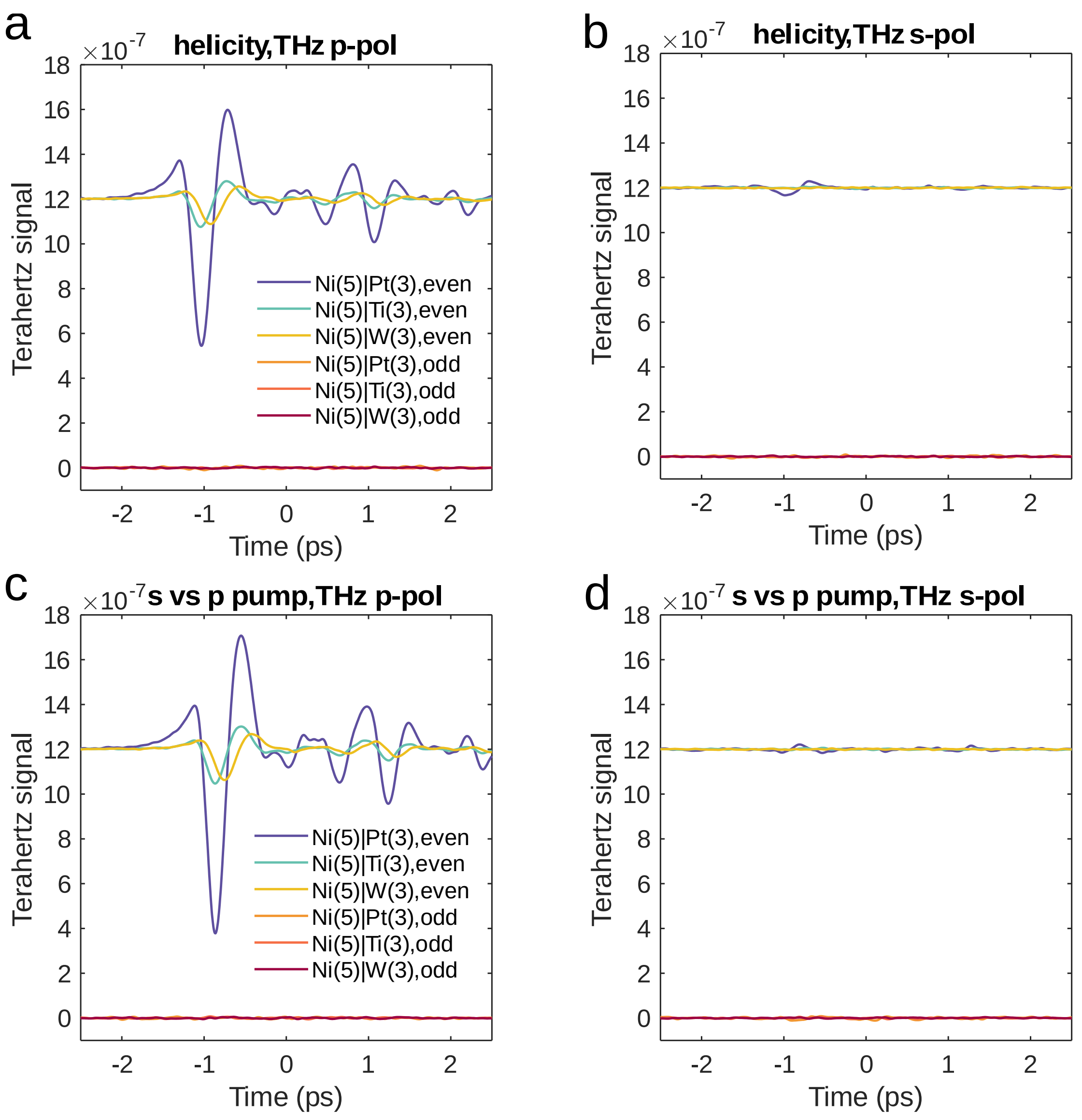

FIGURE S4: Impact of pump polarization. a, **b**, Circular pump polarization. THz-emission signals even (LCP+RCP) and odd (LCP−RCP) with respect to the pump helicity, i.e., left-handed (LCP) and right-handed circular polarization (RCP). The THz emission is polarized along the p-direction (panel a) and s-direction (panel b). **c**, **d**, Linear pump polarization. THz emission signals even (s+p) and odd (s−p) with respect to linear pump polarization (s- and p-polarized). The THz emission is polarized along the p-direction (panel c) and s-direction (panel d), and samples are magnetized along the s-direction. Film thicknesses in nanometers are given as numerals in parenthesis. This figure implies that the THz-emission signal does neither depend on the pump helicity (left- vs right-circular; panels a, b) nor on the linear polarization direction (s vs p; panels c, d).

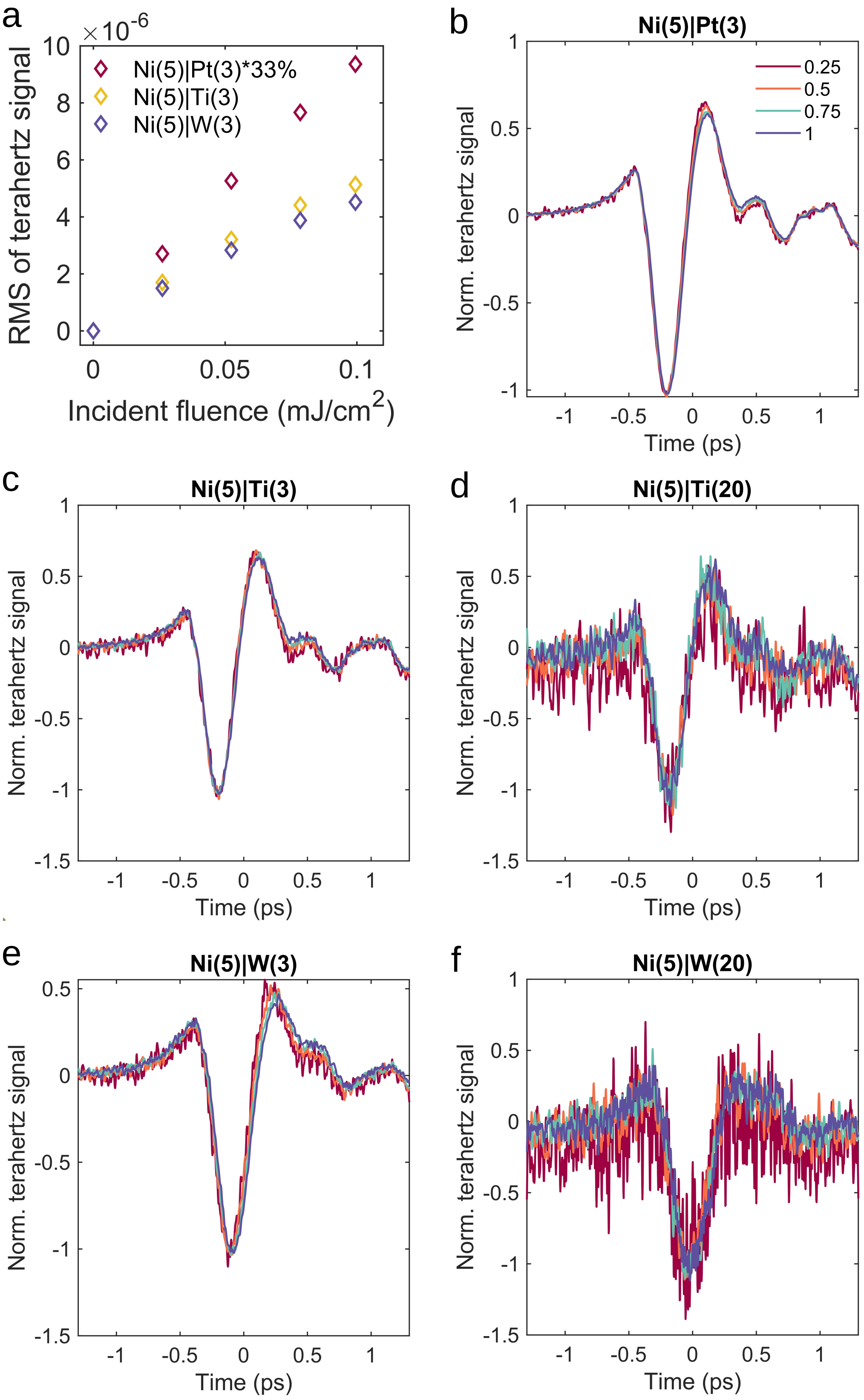

FIGURE S5: Pump-fluence dependence. a, Fluence dependence of the THz-emission signal from Ni capped with Pt, W or Ti. The data was contracted by taking the root mean square (RMS) of the time-domain traces. **b-f**, Normalized THz-emission signals for different pump fluences. The different colors correspond to the fluence levels applied (25%: red, 50%: orange, 75%: cyan and 100%: blue). Film thicknesses in nanometers are given as numerals in parentheses.

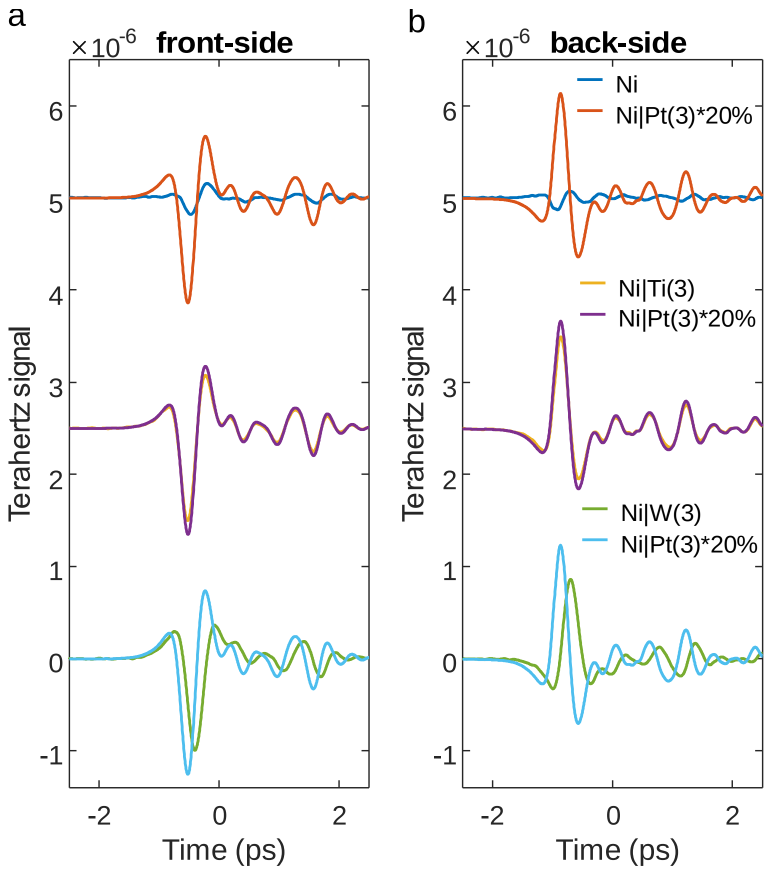

FIGURE S6: Front-side vs back-side pump geometry. a, THz-emission signals from samples pumped from the front side and **b**, the back side. Back-side pumping is defined as the direction where the pump pulse first traverses the substrate before exciting the sample film. It is the standard direction used for all measurements throughout this work. Film thicknesses in nanometers are given as numerals in parenthesis, except for the Ni layers, which are always 5 nm thick.

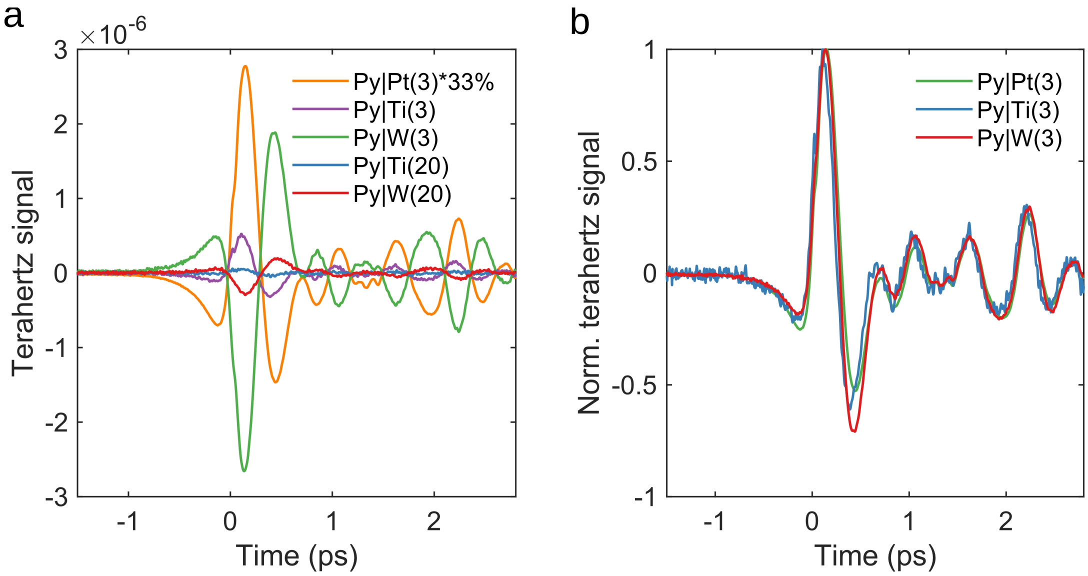

FIGURE S7: THz-emission signals for Py-based samples. a, THz-emission signals from thicker Ti and W layers on Py. **b**, Normalized THz-emission signals of the data shown in Fig. 2a in the main text. Film thicknesses in nanometers are given as numerals in parentheses, except for the Py layers, which are always 5 nm thick.

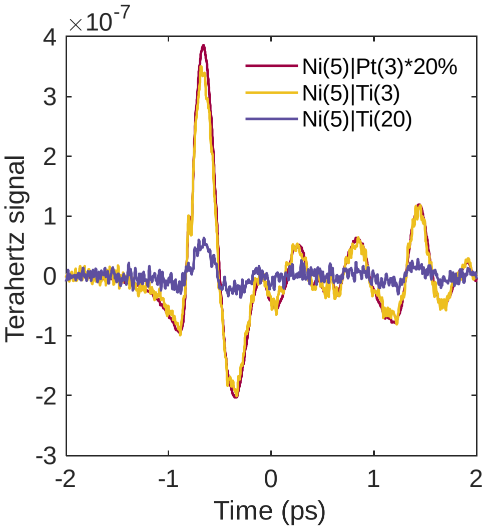

FIGURE S8: Ni|Pt vs Ni|Ti. THz-emission signals from Ni|Pt vs Ni|Ti. Film thicknesses in nanometers are given as numerals in parentheses. Note the rescaling of the Ni|Pt sample waveform.

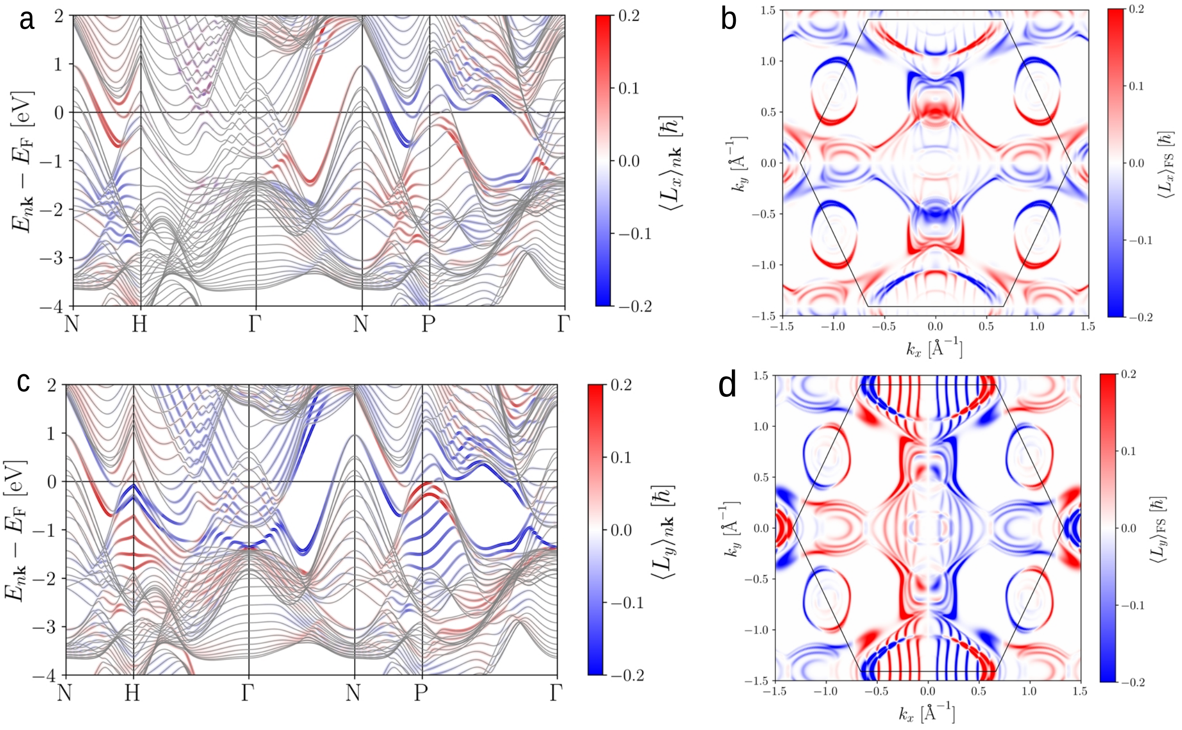

FIGURE S9: *Ab-initio* calculation of the electronic structure and $\mathbf{L}$ texture at the surface of a W thin film. The film consists of 19 layers of W atoms in bcc(110) stacking, and $\mathbf{L}$ is evaluated for the two topmost surface atoms. **a**, Electronic band structure $E_{n\mathbf{k}}$ (grey lines) together with the expectation value of L_x for individual states. **b**, $\mathbf{k}$-space texture of L_x,. **c**, **d**, Same as panels a, b, respectively, but for L_y.

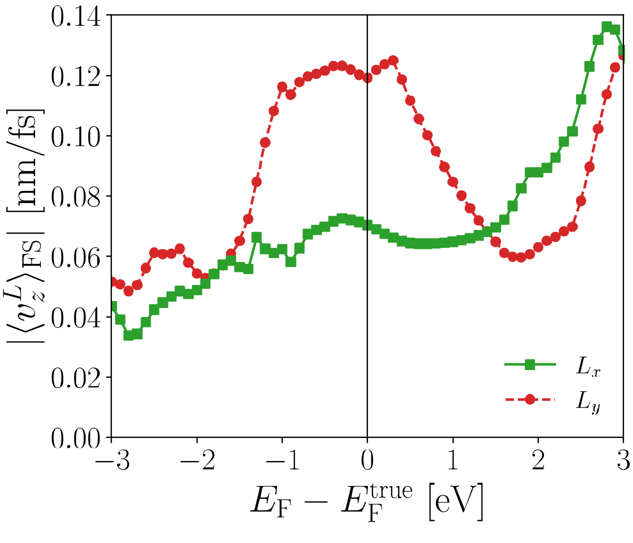

FIGURE S10: Calculated orbital velocity at the Fermi surface. The orbital velocity is calculated for states at the Fermi surface for bulk W in bcc structure for different values of the Fermi energy with respect to the true Fermi energy (see Methods). The coordinate system is defined such that $x \parallel [001]$, $y \parallel [1\bar{1}0]$, $z \parallel [110]$.

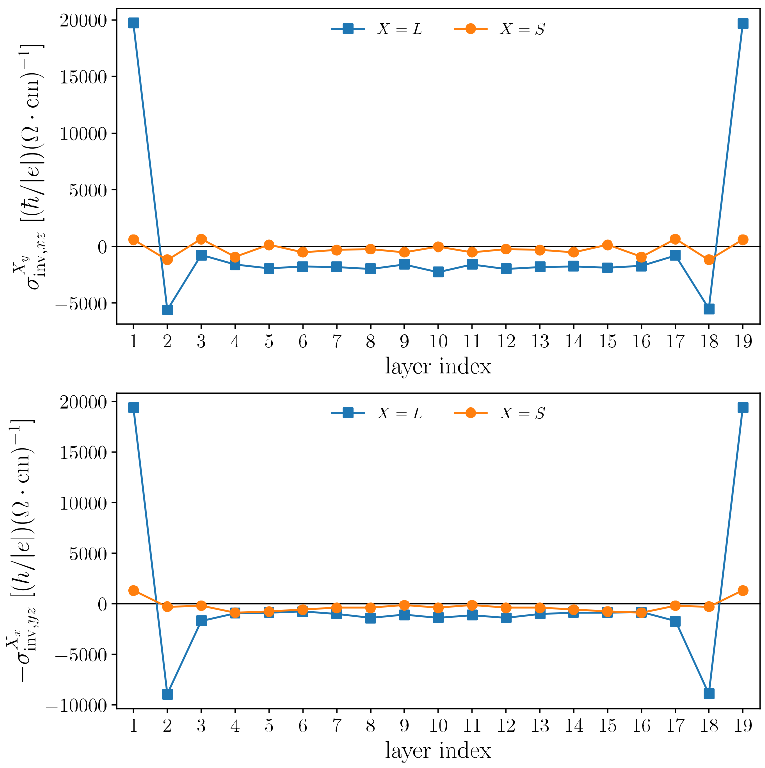

FIGURE S11: *Ab-initio* calculation of the charge current response to an orbital-dependent chemical potential in a W thin film. The film consists of 19 layers of W atoms in bcc(110) stacking. The response is calculated by the Kubo formula (see Methods). The charge-current response is strongly pronounced at the W/vacuum surfaces and has positive sign, which coincides with the sign of the inverse spin Hall effect of Pt. The coordinate system is defined such that $x \parallel [001]$, $y \parallel [1\bar{1}0]$, $z \parallel [110]$.

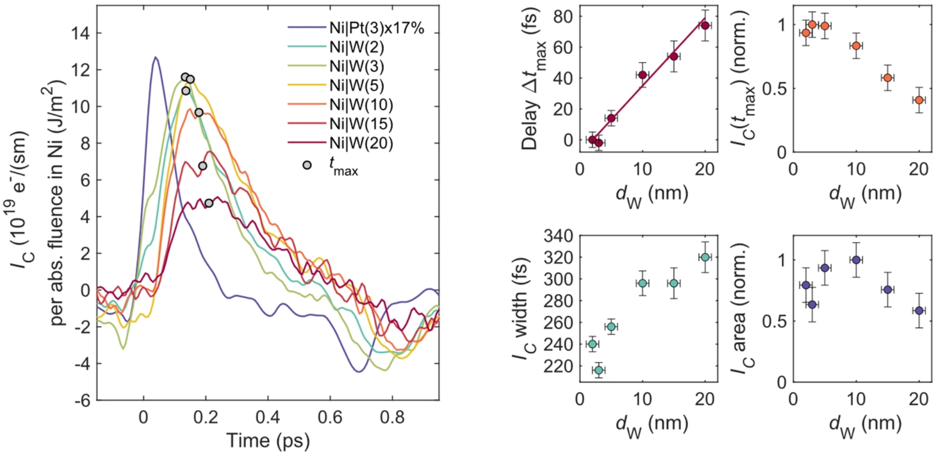

a, Measured data. For details about the error estimates, please refer to the Methods section.

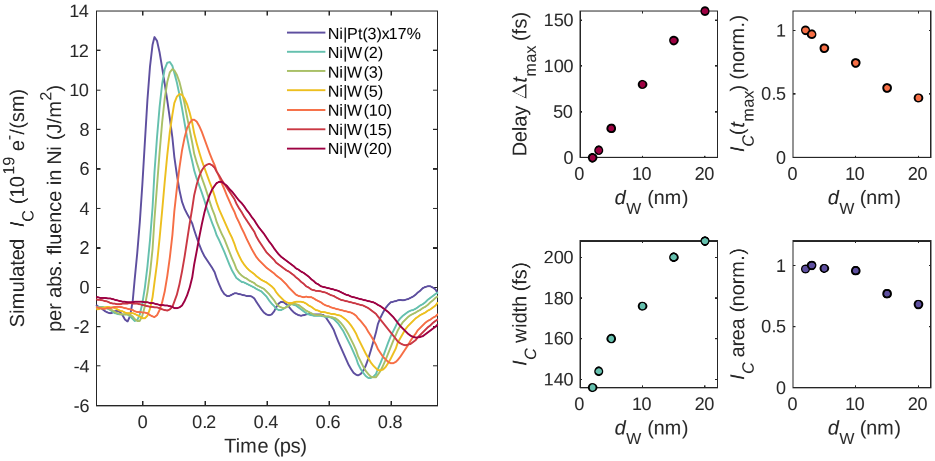

b, Ballistic scenario, v_L = 0.14 nm/fs, L decay length of 80 nm

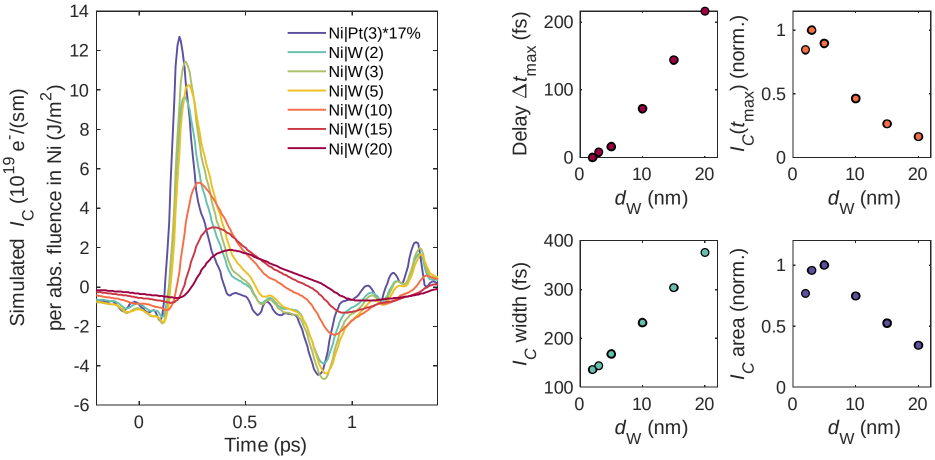

c, Diffusive scenario, v_L = 0.9 nm/fs, L decay length of 1.4 nm

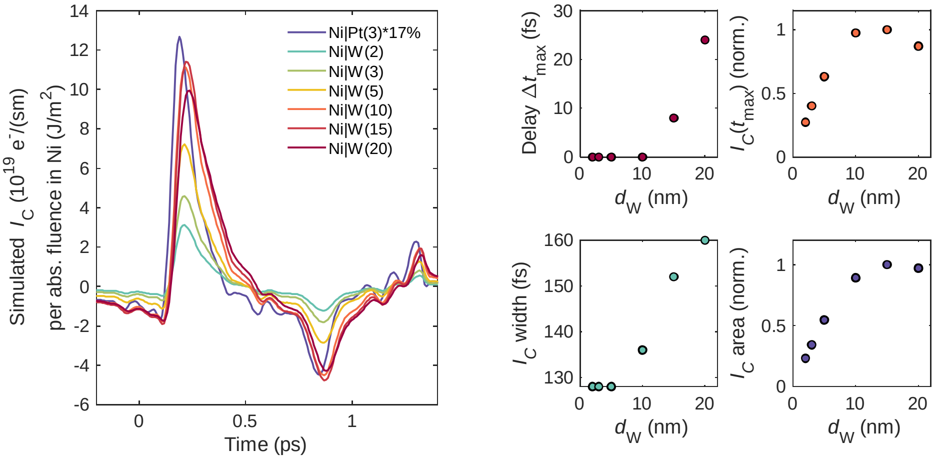

d, Diffusive scenario, v_L = 0.9 nm/fs, L decay length of 20 nm

FIGURE S12: Comparison of modeled charge currents for different propagation regimes. a, Experimental data. Simulations for **b**, ballistic limit and **c**, **d**, diffusive limit. The right-hand side of each panel shows the extracted charge current parameters as defined in Fig. 4 of the main text. Panel d is shown in the next page.

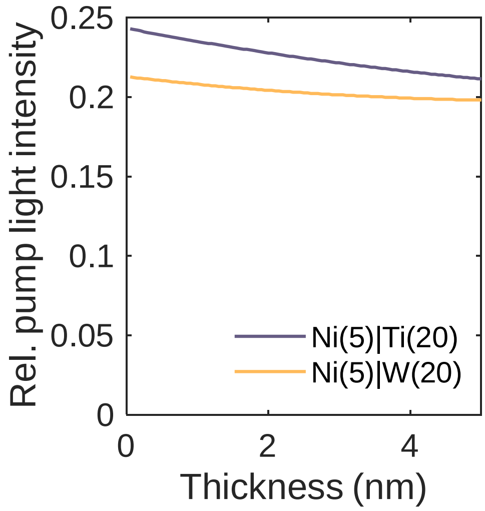

FIGURE S13: Calculated pump-intensity gradient in Ni for Ni(5)|Ti(20) and Ni(5)|W(20) samples, which are the thickest samples measured. However, even in these samples, the pump-light gradient is minor. The calculation is based on a transfer-matrix formalism [5]. Film thicknesses in nanometers are given as numerals in parentheses.

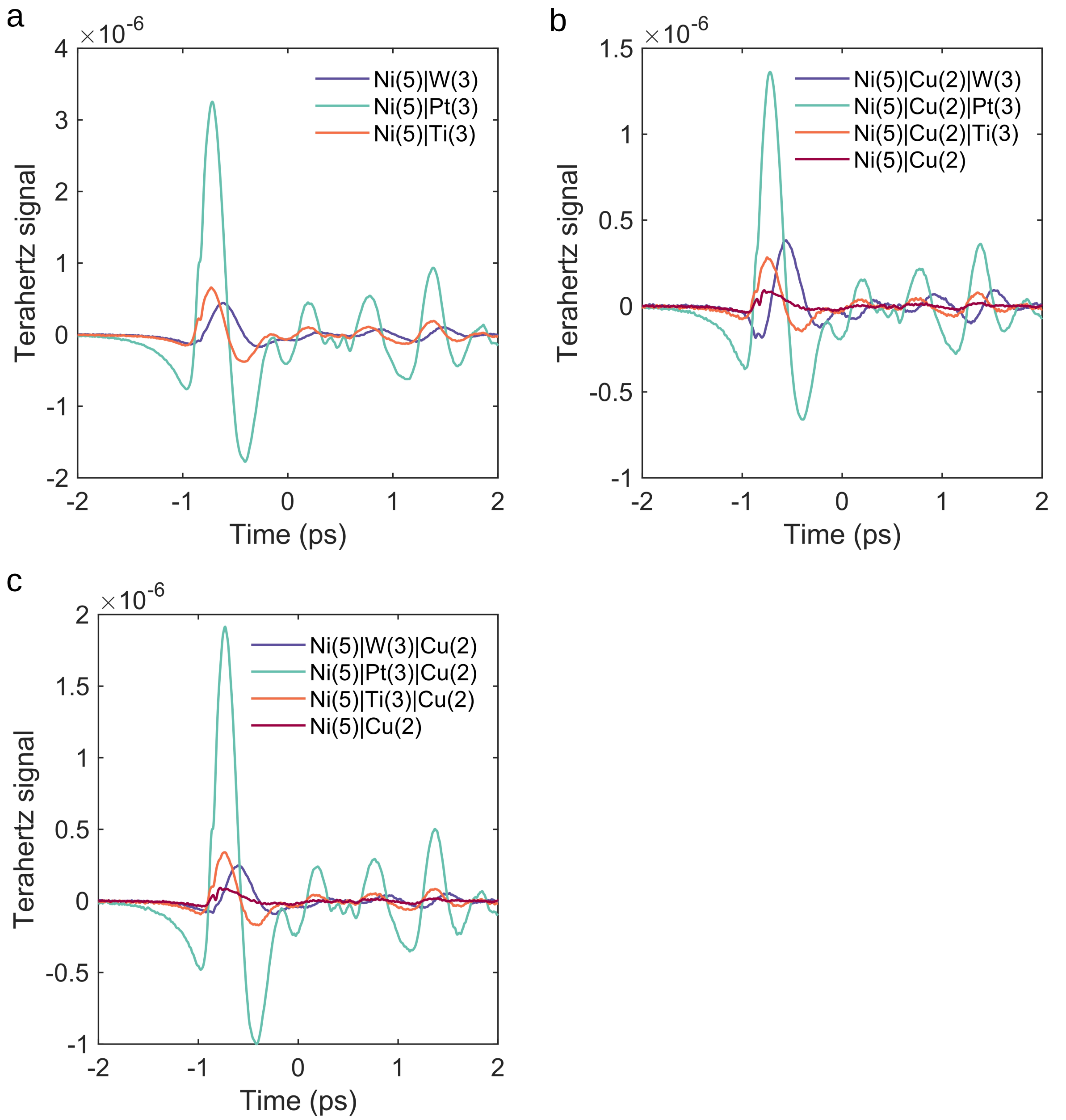

FIGURE S14: Impact of Cu interlayers and capping layers. THz-emission signals from **a**, reference samples without Cu, **b**, samples with Cu intermediate layer and **c**, samples with Cu capping layer. Film thicknesses in nanometers are given as numerals in parentheses.

Sample	Absorptance	Absorbed fluence in the FM layer (mJ/cm^2)	Absorbed fluence in the PM layer (mJ/cm^2)	Conductivity (MS/m)
Glass| Ti(50)	-	-	-	1.6
Glass| Ni(5)|W(20)	0.52	0.06	0.20	5.1
Glass| Ni(5)|Pt(3)	0.63	0.25	0.06	3.6
Glass| Ni(5)|Ti(3)	0.58	0.19	0.10	2.2
Glass| Ni(5)|W(3)	0.58	0.19	0.10	2.1
Glass| Ni(5)|Ti(20)	0.51	0.06	0.20	1.6
Glass| Ni(5)	0.51	0.25	-	1.7
Glass| Py(5)|W(3)	-	-	-	2.2
Glass| Py(5)|Ti(3)	-	-	-	1.5
Glass| Py(5)|Pt(3)	-	-	-	2.5
Glass| Py(5)|W(20)	-	-	-	5.3
Glass| Py(5)	-	-	-	2.4
Glass| Py(5)|Ti(20)	-	-	-	1.2
Glass| Ni(5)|Ti(3)|Cu(2)	0.53	-	-	3.1
Glass| Ni(5)|Pt(3)|Cu(2)	0.54	-	-	4.2
Glass| Ni(5)|W(3)|Cu(2)	0.58	-	-	4.0
Glass| Ni(5)|Cu(2)	0.52	-	-	4.5
Glass| Ni(5)|Cu(2)|Ti(3)	0.56	-	-	3.4
Glass| Ni(5)|Cu(2)|Pt(3)	0.54	-	-	3.7
Glass| Ni(5)|Cu(2)|W(3)	0.57	-	-	3.4
Glass| Ni(5)|W(15)	0.54	0.07	0.19	4.7
Glass| Ni(5)|W(10)	0.57	0.10	0.18	4.2
Glass| Ni(5)|W(5)	0.63	0.16	0.15	3.6
Glass| Ni(5)|W(2)	0.60	0.22	0.08	2.9
Si| Ni(5)|W(3)	-	-	-	2.9
Si| Ni(5)|Ti(20)	-	-	-	1.6
Si| Ti(50)	-	-	-	1.5
Si| Ni(5)|Pt(3)	-	-	-	3.4
Si| Ni(5)|W(20)	-	-	-	4.3
Si| Ni(5)	-	-	-	3.3
Si| Ni(5)|Ti(3)	-	-	-	2.3

Table S1. Optical and properties of all studied samples. To obtain the absorbed fluence in the FM and PM layer, we assume imaginary parts of the dielectric constants (wavelength 800 nm) of 22.07 for Ni, 9.31 for Pt, 19.41 for Ti and 19.71 for W [6]. All films are additionally capped with 4 nm SiO_2. In the first column, film thicknesses in nanometers are given as numerals in parentheses.

Supplementary Information References

1. Zhang, W., P. Maldonado, Z. Jin, T.S. Seifert, J. Arabski, G. Schmerber, E. Beaurepaire, M. Bonn, T. Kampfrath, P.M. Oppeneer D. Turchinovich, *Ultrafast terahertz magnetometry.* Nat Commun, 2020. **11**(1): p. 4247.
2. Rouzegar, R., L. Brandt, L. Nadvornik, D.A. Reiss, A.L. Chekhov, O. Gueckstock, C. In, M. Wolf, T.S. Seifert, P.W. Brouwer, G. Woltersdorf T. Kampfrath, *Laser-induced terahertz spin transport in magnetic nanostructures arises from the same force as ultrafast demagnetization.* Physical Review B, 2022. **106**(14): p. 144427.
3. Seifert, T.S., N.M. Tran, O. Gueckstock, S.M. Rouzegar, L. Nadvornik, S. Jaiswal, G. Jakob, V.V. Temnov, M. Münzenberg, M. Wolf, M. Kläui T. Kampfrath, *Terahertz spectroscopy for all-optical spintronic characterization of the spin-Hall-effect metals Pt, W and Cu80Ir20.* Journal of Physics D: Applied Physics, 2018. **51**(36): p. 364003.

4. Nádvorník, L., M. Borchert, L. Brandt, R. Schlitz, K.A. de Mare, K. Výborný, I. Mertig, G. Jakob, M. Kläui S.T. Goennenwein, *Broadband terahertz probes of anisotropic magnetoresistance disentangle extrinsic and intrinsic contributions.* Physical Review X, 2021. **11**(2): p. 021030.
5. Zak, J., E. Moog, C. Liu S. Bader, *Universal approach to magneto-optics.* Journal of Magnetism and Magnetic Materials, 1990. **89**(1-2): p. 107-123.
6. Ordal, M.A., L.L. Long, R.J. Bell, S.E. Bell, R.R. Bell, R.W. Alexander, Jr. C.A. Ward, *Optical properties of the metals Al, Co, Cu, Au, Fe, Pb, Ni, Pd, Pt, Ag, Ti, and W in the infrared and far infrared.* Appl Opt, 1983. **22**(7): p. 1099-20.
