# Supplementary material for: Time-domain observation of ballistic orbital-angular-momentum currents with giant relaxation length in tungsten
Source: Nat Nanotechnol. 2023 Aug 7;18(10):1132–8. doi: 10.1038/s41565-023-01470-8 (PMC10575790; doi:10.1038/s41565-023-01470-8)
Supplement: Supplementary file 2 — Reporting Summary [file 41565_2023_1470_MOESM2_ESM.pdf]

Corresponding author(s): Tom Seifert

Lasing Reporting Summary

Nature Research wishes to improve the reproducibility of the work that we publish. This form is intended for publication with all accepted papers reporting claims of lasing and provides structure for consistency and transparency in reporting. Some list items might not apply to an individual manuscript, but all fields must be completed for clarity.

For further information on Nature Research policies, including our data availability policy, see Authors & Referees.

Experimental design

Please check: are the following details reported in the manuscript?

1. Threshold

Plots of device output power versus pump power over a wide range of values indicating a clear threshold

☐ Yes
☒ No

not subject of this study

2. Linewidth narrowing

Plots of spectral power density for the emission at pump powers below, around, and above the lasing threshold, indicating a clear linewidth narrowing at threshold

☐ Yes
☒ No

not subject of this study

Resolution of the spectrometer used to make spectral measurements

☐ Yes
☒ No

not subject of this study

3. Coherent emission

Measurements of the coherence and/or polarization of the emission

☒ Yes
☐ No

Figure S3

4. Beam spatial profile

Image and/or measurement of the spatial shape and profile of the emission, showing a well-defined beam above threshold

☐ Yes
☒ No

not subject of this study

5. Operating conditions

Description of the laser and pumping conditions
Continuous-wave, pulsed, temperature of operation

☒ Yes
☐ No

Section "Experimental details" in the main text

Threshold values provided as density values (e.g. W cm^{-2} or J cm^{-2}) taking into account the area of the device

☐ Yes
☒ No

not subject of this study

6. Alternative explanations

Reasoning as to why alternative explanations have been ruled out as responsible for the emission characteristics
e.g. amplified spontaneous, directional scattering; modification of fluorescence spectrum by the cavity

☐ Yes
☒ No

not subject of this study

7. Theoretical analysis

Theoretical analysis that ensures that the experimental values measured are realistic and reasonable
e.g. laser threshold, linewidth, cavity gain-loss, efficiency

☐ Yes
☒ No

not subject of this study

8. Statistics

Number of devices fabricated and tested

☐ Yes
☒ No

not subject of this study

Statistical analysis of the device performance and lifetime (time to failure)

☐ Yes
☒ No

not subject of this study

March 2018
